# In silico prediction and structure-based multitargeted molecular docking analysis of selected bioactive compounds against mucormycosis

**DOI:** 10.1186/s42269-022-00704-4

**Published:** 2022-01-31

**Authors:** Premnath Madanagopal, Nagarjun Ramprabhu, Rahul Jagadeesan

**Affiliations:** Department of Biotechnology, Alagappa College of Technology, Anna University, Chennai, India

**Keywords:** Mucormycosis, Covid-19, Virtual screening, ADMET, Black fungus, Molecular docking

## Abstract

**Background:**

During the second wave of the COVID-19 pandemic, an unusual increase in cases of mucormycosis was observed in India, owing to immunological dysregulation caused by the SARS-CoV-2 and the use of broad-spectrum antibiotics, particularly in patients with poorly controlled diabetes with ketoacidosis to have contributed to the rise, and it has been declared an epidemic in several states of India. Because of the black colouring of dead and dying tissue caused by the fungus, it was dubbed "black fungus" by several Indian media outlets. In this study, attempts were taken to unmask novel therapeutic options to treat mucormycosis disease. *Rhizopus* species is the primary fungi responsible for 70% of mucormycosis cases.

**Results:**

We chose three important proteins from the *Rhizopus delemar* such as CotH3, Lanosterol 14 alpha-demethylase and Mucoricin which plays a crucial role in the virulence of Mucorales. Initially, we explored the physiochemical, structural and functional insights of proteins and later using AutoDock Vina, we applied computational protein–ligand binding modelling to perform a virtual screening around 300 selected compounds against these three proteins, including FDA-approved drugs, FDA-unapproved drugs, investigational-only drugs and natural bioactive compounds. ADME parameters, toxicity risk and biological activity of those compounds were approximated via in silico methods. Our computational studies identified six ligands as potential inhibitors against *Rhizopus delemar*, including 12,28-Oxamanzamine A, vialinin B and deoxytopsentin for CotH3; pramiconazole and saperconazole for Lanosterol 14 alpha-demethylase; and Hesperidin for Mucoricin. Interestingly, 12,28-Oxamanzamine A showed a maximum binding affinity with all three proteins (CotH3: − 10.2 kcal/mol Lanosterol 14 alpha-demethylase: − 10.9 kcal/mol Mucoricin: − 8.6 kcal/mol).

**Conclusions:**

In summary, our investigation identified 12,28-Oxamanzamine A, vialinin B, deoxytopsentin, pramiconazole, saperconazole and hesperidin as potent bioactive compounds for treating mucormycosis that may be considered for further optimisation techniques and in vitro and in vivo studies.

**Supplementary Information:**

The online version contains supplementary material available at 10.1186/s42269-022-00704-4.

## Background

Mucormycosis, a particularly vicious disease currently gaining popularity due to the rising number of cases, is a disease whose ferocity which humanity has not fully understood (Nicolás et al. 2020). Mucorales is one of the most densely studied orders of fungi, and the fungal infections or mycoses caused by this order are referred to as mucormycosis. Many in this order were classified as harmless. Still, later after they caused certain invasive diseases, humans were able to conclude that this is a perilous group of species and that the taxonomy of this fungi is so mixed up that many cannot comprehend. Phycomycosis, zygomycosis, entomophthoramycosis were used interchangeably to describe mucormycosis, regarding which species were studied at that particular period (Sugar 1992; Reid et al. 2020; Lehrer et al. 1980; Ibrahim et al. 2012). Mucorales have always been notorious amongst other fungal orders, and identification of the causal organism is crucial in constructing a cure for the disease caused by it (Balajee et al. 2009; Walther et al. 2020). This complexity has invariably caused a backlog in synthesising a particular drug that can cure the disease. But a cure isn't child's play, rather an arduous task. *Rhizopus* species are the most common fungi in the order of Mucorales responsible for over 70% of mucormycosis cases (Gebremariam et al. 2014). The occurrence of mucormycosis has been about 0.005–1.7 per million population. Still, in countries like India, the prevalence is as high as 0.14 per 1000 people, about 80 times higher than the world incidence rate (Singh et al. 2021). Mucormycosis can occur as three variants: rhinocerebral (sinus and brain) mucormycosis, pulmonary (lung) mucormycosis, gastrointestinal or cutaneous (skin) mucormycosis (Additional file 1: Table S1).

Mucormycosis has specific comorbidities, making it even riskier to contract the disease, leading to a higher mortality rate. These factors are neutropenia, excessive iron, protein-calorie malnutrition (PCM) and diabetic ketoacidosis. SARS-Cov-2, in addition to mucormycosis, is a fatal combination that has caused a considerable number of deaths, particularly in India (Singh et al. 2021; Agrawal et al. 2020; Hong et al. 2013; Afroze et al. 2017; Gangadharan et al. 2017; Kubin et al. 2019; Chander et al. 2018). Doctors discovered that this fungus only infects people with highly impaired immune systems, such as COVID-19 patients with diabetes or high uncontrolled blood sugar levels following recovery (Garg et al. 2021). It was observed that the possible reason for this infection is the indiscriminate use of steroids for the treatment of COVID-19 patients. It was cited that when the body's system fights against a virus, the use of steroids in COVID-19 patients reduced inflammation within the lungs. Still, uncontrolled use of steroids doses also reduced immunity and elevated blood sugar levels because of less physical activity in diabetic and non-diabetic people, thus increasing the chance of infecting with mucormycosis (BBC News 2021).

Currently, few drugs like amphotericin B, posaconazole and rarely isavuconazole or triazole are suggested for treatment (Naqvi et al. 2020). However, there is no specific therapeutics that is available for mucormycosis and thus, further exploration into existing drugs (drug repurposing), as well as natural compounds against mucormycosis, is required. Even in today's scientific world, creating a new drug is an intricate process requiring a vast number of resources and workforce, and so, the use of in silico techniques has become an important aspect of the drug development process. This is mostly due to their ability to influence the entire drug development process, finding and discovering new prospective medications while reducing cost and time (Brogi et al. 2020).

This study attempts structure-based computational screening of the bioactive compounds against potential protein targets of *Rhizopus delemar* (Table 1). we worked on three proteins that were found to be very important when it comes to the virulence of mucormycosis disease. These three proteins are discussed below:

1. CotH3: CotH3 proteins were widely present in Mucorales and absent in non-invasive pathogens. This spore coat protein homolog (CotH3) acts as a fungal ligand for host cell GRP78 and mediates pathogenic host-cell interactions. The presence of CotH3 in Mucorales also explained why DKA patients with high GRP78 levels are more susceptible to mucormycosis (Gebremariam et al. 2014).
2. Lanosterol 14 alpha-demethylase: It plays a vital role in the biosynthesis of sterol in fungi and is an essential enzyme in the fungal life cycle (Sheng et al. 2009).
3. Mucoricin: It is a ricin-like toxin important in the pathogenesis of mucormycosis. Also, it is a Ribosome-inactivating protein that promotes vascular permeability and induces both necrosis and apoptosis of host cells (Soliman et al. 2021).

**Table 1:** Predicted gene ontology (GO) terms by CI-TASSER

Proteins	Molecular function (MF)	Biological process (BP)	Cellular component (CC)
CotH3	Phosphatidylinositol kinase activity (GO:0,052,742) Purine ribonucleoside triphosphate binding (GO:0,035,639) Hydrolase activity, hydrolysing O-glycosyl compounds (GO:0,004,553)	Single-organism process (GO:0,044,699) Asexual sporulation (GO:0,030,436)	Cell part (GO:0,044,464)
Lanosterol 14 alpha-demethylase	Oxidoreductase activity (GO:0,016,491) Monooxygenase activity (GO:0,004,497) Heme binding (GO:0,020,037) Sterol 14-demethylase activity (GO:0,008,398) Iron ion binding (GO:0,005,506)	Single-organism metabolic process (GO:0,044,710) Biosynthetic process (GO:0,009,058) Lipid metabolic process (GO:0,006,629) Oxidation–reduction process (GO:0,055,114)	Membrane (GO:0,016,020)
Mucoricin	Catalytic activity (GO:0,003,824) Carbohydrate binding (GO:0,030,246)	Carbohydrate metabolic process (GO:0,005,975) Cellular process (GO:0,009,987)	Cell part (GO:0,044,464)

**Fig. 1:**
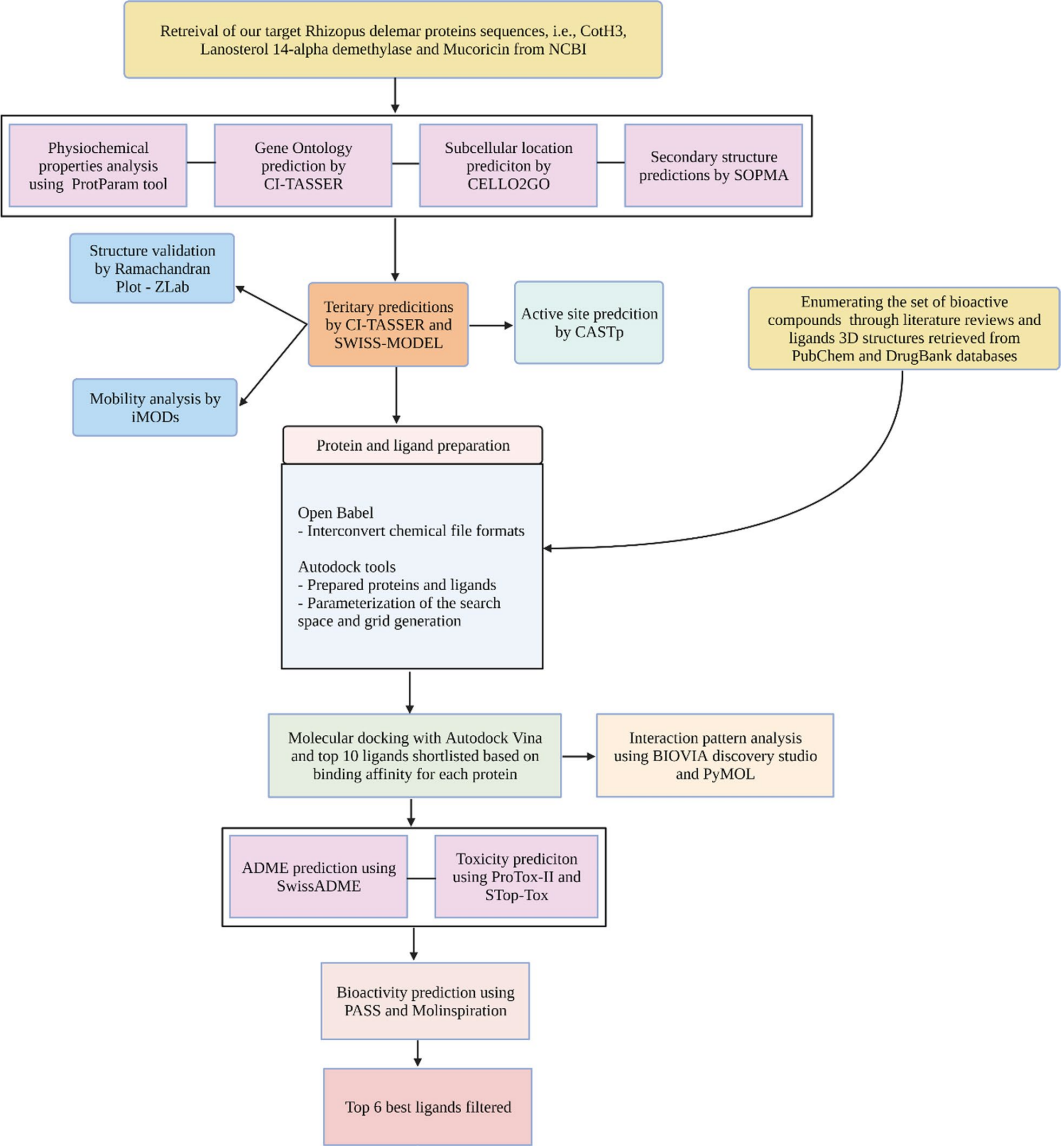
Flowchart depicting the workflow of our structure-based virtual screening

These crucial proteins (CotH3, Lanosterol 14 alpha-demethylase and Mucoricin) require a thorough examination of their structure and function, which will bring unique insights into the development of an effective, low-cost medicine with minimal side effects. Therefore, the current study aims to collect 300 compounds [FDA approved, FDA unapproved, investigational-only, natural compounds] that exhibit antiviral, antifungal, antibacterial and antimicrobial properties have been identified through different literature reviews, and it was screened against CotH3, Lanosterol 14 alpha-demethylase and Mucoricin by applying several in silico tools, viz., protein modelling, binding pocket prediction, molecular docking, ADME and drug-likeness screening, bioactivity prediction and toxicity prediction (Fig. 1).

## Methods

### Proteins sequence retrieval

Proteins used in this study are the ones that are majorly involved in mucormycosis. The NCBI protein database (Home - Protein - NCBI (n.d.). 2021) was searched for the sequence retrieval of the *Rhizopus delemar* spore coat protein homologs CotH3 (ACCESSION: EIE87171 region: CotH), cytochrome P450 enzyme Lanosterol 14 alpha-demethylase (ACCESSION: EIE87079) and ricin-like toxin Mucoricin (ACCESSION: EIE81863) (Ma et al. 2009).

### Analysis of physicochemical properties and subcellular localisation

Various physicochemical properties of the CotH3, Lanosterol 14 alpha-demethylase and Mucoricin were calculated using ExPasy's ProtParam tool (Gasteiger et al. 2005). Molecular weight, theoretical pI, grand average of hydropathy (GRAVY), half-life, aliphatic index (AI), instability index and amino acid composition were calculated. For understanding protein function, it is essential to find out the subcellular localisation of proteins. CELLO2GO server was used for this purpose (Yu et al. 2014).

### Secondary structure prediction

The secondary structure features of the protein such as α helix, 3_10_ helix, Pi helix, Beta Bridge, Extended strand, Bend region, Beta turns, Random coil, Ambiguous states and other states were determined using a self-optimised prediction method (SOPMA) (NPS@ 2021).

### Tertiary structure prediction

All three proteins (i.e. CotH3, Lanosterol 14 alpha-demethylase, Mucoricin) were subjected to 3D modelling. CotH3 was modelled via SWISS-MODEL (Waterhouse et al. 2018), and Lanosterol 14 alpha-demethylase and Mucoricin were modelled via C-I-TASSER (Contact-Guided Protein Structure Prediction) (Zheng et al. 2021). The SWISS-MODEL web server automatically calculates the QMEAN scoring function to estimate the local and the global model quality based on the geometry, the interactions and the solvent potential of the protein model. It also provides the z-score ranging from 0 to 1, compared with the expected value for any structure. C-I-TASSER uses highly accurate deep learning-based predicted contacts to guide its replica-exchange Monte Carlo (REMC) simulations to generate models.

### Active site prediction and mobility analysis

The Computed Atlas of Surface Topography of proteins (CASTp) 3.0 was used to predict probable binding pockets of the proteins (Tian et al. 2018). CASTp is an online server used to identify and determine the binding sites, surface structural pockets, area, shape and volume of every pocket and internal cavities of proteins. It could also be used to assess the number, boundary of mouth openings of every pocket, molecular reachable surface and area. The modelled 3D protein was submitted on the server, and the necessary amino acids for binding interactions were predicted. iMODs server (López-Blanco et al. 2014) was used to predict the extent and direction of the inherent motions of studied proteins. It represents the collective motion of proteins by evaluating the normal modes (NMA) in internal coordinates predicting properties such as deformability, mobility profiles, eigenvalues, variance and covariance map.

### Protein preparation and ligand preparation

The target proteins were prepared before starting the docking processes. It was done with the help of AutoDockTools (ADT), part of MGLTools (Morris et al. 2009). Proteins were prepared by correcting bonds, removing unrelated chemical complexes, eliminating water molecules and HETATM groups, adding hydrogen bonds, filling the missing side-chain atoms, adding the necessary charges and atom types, and saving in PDBQT format in preparation for molecular docking.

As already mentioned, about 300 compounds [FDA approved, FDA unapproved, investigational-only, natural compounds] that exhibit antiviral, antifungal, antibacterial and antimicrobial properties have been identified through different literature reviews (Parsaeimehr and Lutzu 2016; Vila et al. 2013; Vengurlekar et al. 2012). The compounds were selected based on experimental evidence of different enzymatic and assays. The SDF structures of those compounds were retrieved from the DrugBank (Wishart et al. 2008) and PubChem database (Kim et al. 2016). The compounds were converted to PDB chemical format using the Open Babel program (O’Boyle et al. 2011). Open Babel is a software mainly used to interconvert chemical file formats. Further, compounds were prepared and converted to the dockable PDBQT format using Autodock tools.

### Molecular docking

Molecular docking is a helpful tool for performing virtual screening on various compounds and inferring how the ligands bind to their targets. Docking of the ligands to the targeted proteins and determination of binding affinities were carried out using AutodockVina (Trott and Olson 2010). In this study, proteins were kept rigid, and ligands were kept flexible. Intermediary steps, such as PDBQT files for proteins and ligands preparation and grid box creation, were completed using AutoDock Tools. The box type and grid box parameters are given in Table 2.

**Table 2:** AutoDock mediated docking parameters like box type and grid box information for our target proteins

Proteins	Box type	X	Y	Z
CotH3	Cube	27.51	47.54	31.08
Lanosterol 14 alpha-demethylase	Cube	83.377	79.808	80.265
Mucoricin	Cube	62.464	58.547	57.827

### Visualisation and molecular interactions

The molecular interactions between the proteins and ligands with the least energy were viewed with Discovery Studio Visualizer, BIOVIA, 2021(Biovia 2021) and PyMOL software (Schrödinger 2021).

### ADME analysis and toxicity prediction

The drug-likeness properties of the final lead compounds were calculated by using SwissADME (Daina et al. 2017). Absorption, distribution, metabolism, excretion (ADME) properties were used to eliminate inappropriate compounds. The predicted result from SwissADME consists of physiochemical properties, lipophilicity, water-solubility, pharmacokinetics, drug-likeness and bioavailability Score.

We also performed toxicity prediction of those final compounds to check and verify minor toxic drugs for human use. The analyses were performed using ProTox-II (Banerjee et al. 2018) and STopTox (Borba et al. 2020). ProTox is a useful tool to identify any undesirable toxic properties of our molecules. The prediction was based on functional group similarity for the query molecules with the in vitro and in vivo contained in the database. StopTox is used to assess the potential of chemicals to cause acute toxicity, and it is done by implementing QSAR models. Toxic properties such as LD50 values in mg/kg, toxicity class, acute inhalation toxicity, acute oral toxicity, acute dermal toxicity, eye irritation and corrosion, skin sensitisation, skin irritation and corrosion were determined.

### Bioactivity prediction

The PASS (prediction of activity spectra for substances) program (Lagunin et al. 2000) is an online server to evaluate the overall biological potential of a compound based on its structure–activity relationship. It predicts the appropriate pharmacological effects by comparing the desired structure with a training set that includes more than 205,000 compounds, revealing more than 7200 biological activities. The results of PASS prediction were summarised as a list of probable biological activities, with a probability of being active (Pa) and a probability of being inactive (Pi). Also, the pharmacokinetic properties and bioactivity scores were calculated by the Molinspiration tool (Molinspiration Cheminformatics 2021). Bioactivity scores of the compounds were predicted for drug targets, including enzymes, nuclear receptors, kinase inhibitors, G-protein coupled receptor ligands and ion channel modulators.

## Results

### Analysis of physicochemical properties and subcellular localisation

The physicochemical characteristics and subcellular location of our target proteins are presented in Table 3. The molecular weight of the proteins ranges from 17.1 to 57.8 kDa. The isoelectric points were predicted between 4.22 and 6.65, suggesting that the proteins are acidic. The aliphatic index is in the range of 70.04–88.9, indicating that these proteins are thermally stable and contain a high amount of hydrophobic amino acids. The negative GRAVY values suggesting that these proteins will have a good interaction with water. The localisation of the CotH3, Lanosterol 14 alpha-demethylase and Mucoricin were predicted as extracellular, plasma membrane and cytoplasmic, respectively.

**Table 3:** Physicochemical property and subcellular location analysis of target proteins

Proteins	Physiochemical parameters						Localisation
	Formula	Number of amino acids	Molecular weight (g/mol)	Theoretical pI	Aliphatic index	GRAVY	
CotH3	C_1333_H_1953_N_337_O_403_S_9_	258	29,435.73	4.35	70.04	− 0.409	Extracellular
Lanosterol 14 alpha-demethylase	C_2611_H_4043_N_681_O_737_S_21_	510	57,439.2	6.65	88.9	− 0.109	Plasma membrane
Mucoricin	C_760_H_1149_N_199_O_242_S_6_	147	17,138.03	4.22	79.59	− 0.547	Cytoplasmic

### Secondary structure prediction

Results showed that CotH3 had 40.31% (104 residues) alpha helix, 13.95% (36 residues) extended strand, 2.71% (7 residues) beta turn and 43.02% (111 residues) random coil, while Lanosterol 14 alpha-demethylase showed to have 49.80% (254 residues) alpha helix, 10.78% (55 residues) extended strand, 3.14% (16 residues) beta turn and 36.27% (185 residues) random coil. Similarly, Mucoricin exhibited 5.44% (8 residues) alpha helix, 40.14% (59 residues) extended strand, 14.97% (22 residues) beta turns and 39.46% (58 residues) random coil.

### Protein modelling and structure assessment

**Fig. 2:**
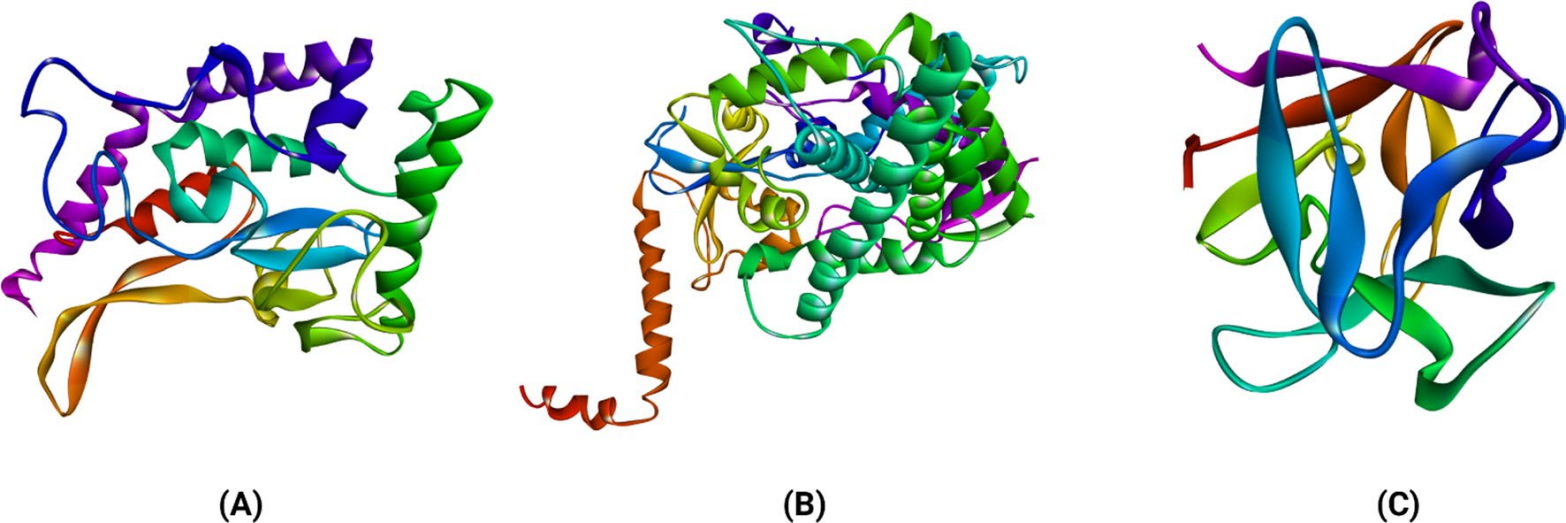
Cartoon representation of structures of modelled *Rhizopus delemar* proteins CotH3 (**a**), Lanosterol 14 alpha-demethylase (**b**) and Mucoricin (**c**)

**Fig. 3:**
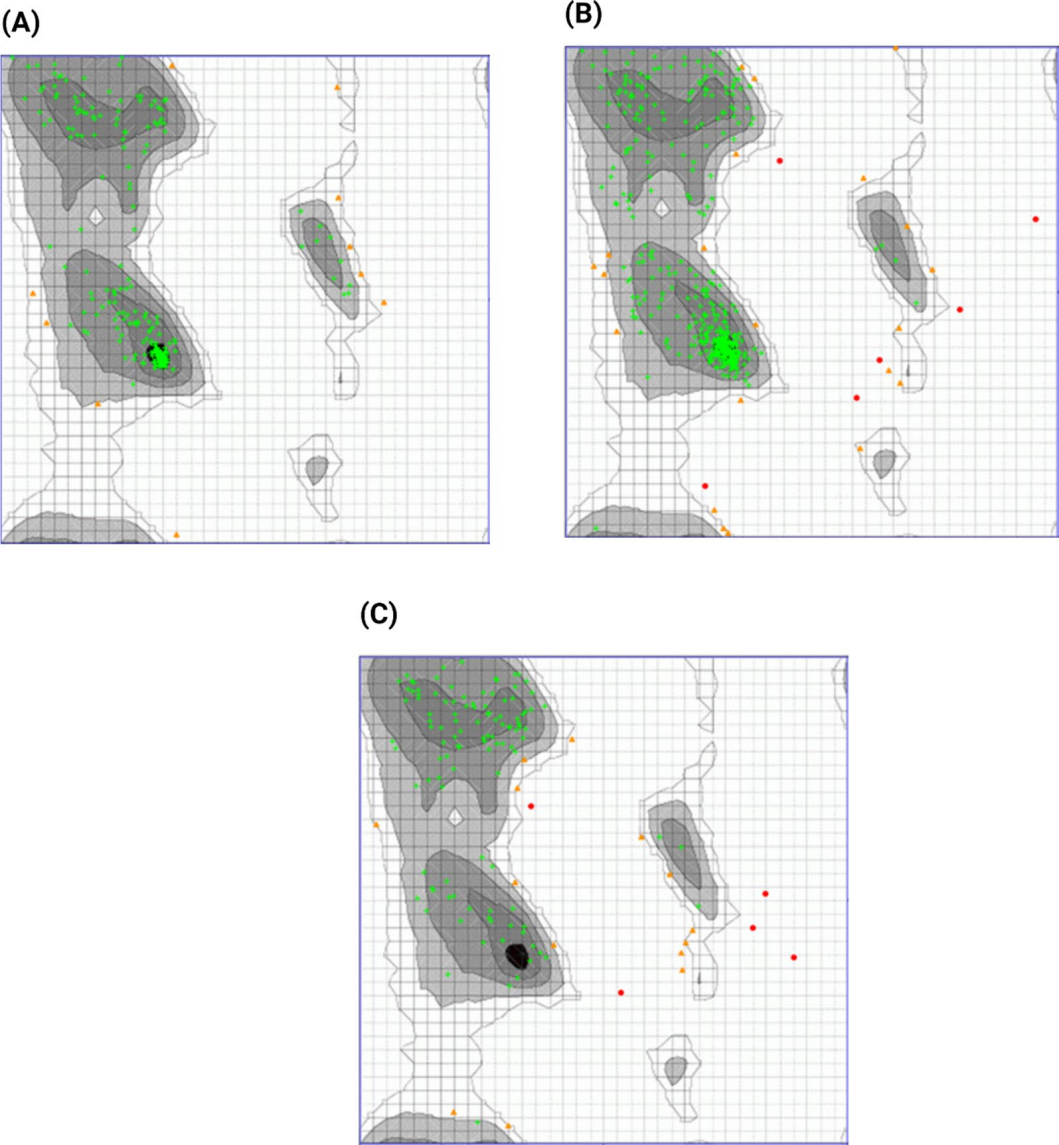
Ramachandran plot of CotH3 protein structure showing the percentage of residues in the highly Preferred observations shown as GREEN Crosses: 217 (95.595%), preferred observations shown as BROWN Triangles: 10 (4.405%) and questionable observations shown as RED Circles: 0 (0.000%) (**a**). Ramachandran plot of Lanosterol 14 alpha-demethylase protein structure showing the percentage of residues in the highly preferred observations shown as GREEN Crosses: 418 (93.933%), preferred observations shown as BROWN triangles: 21 (4.719%) and questionable observations shown as RED circles: 6 (1.348%) (**b**). Ramachandran plot of Mucoricin protein structure showing the percentage of residues in the highly preferred observations shown as GREEN crosses: 113 (85.606%), preferred observations shown as BROWN triangles: 14 (10.606%) and questionable observations shown as RED Circles: 5 (3.788%) (**c**)

The protein modelling for the CotH3 protein was performed using the SWISS-MODEL web server (Waterhouse et al. 2018) (Fig. 2). Crystal structure of *Bacillus cereus* CotH kinase (PDB ID: 5JD9) (Nguyen et al. 2016) was the template lead obtained with 91% sequence coverage with Global Model Quality Estimation (GMQE) value 0.59. The GMQE values are usually between 0 and 1, and higher the number, higher the reliability of the predicted structure. This was used as a template to build a three-dimensional model of the CotH3 protein of *Rhizopus delemar.* The protein structure of Lanosterol 14 alpha-demethylase and Mucoricin were predicted by CI-TASSER (Fig. 2). For each protein, five models were generated, and the model with the highest C-score was selected as the best one and used for further analysis. The drug design process requires the target protein's three-dimensional structure's correctness, quality and reliability. That can be determined by using the ZLab server (Anderson et al. 2005) to develop a Ramachandran plot, which displays allowed, and the disallowed regions regarding backbone dihedrals of protein residues (Fig. 3). The essential condition of being a good quality model is having more than 85–90% of residues in allowed regions.

### Active site prediction and mobility analysis

**Fig. 4:**
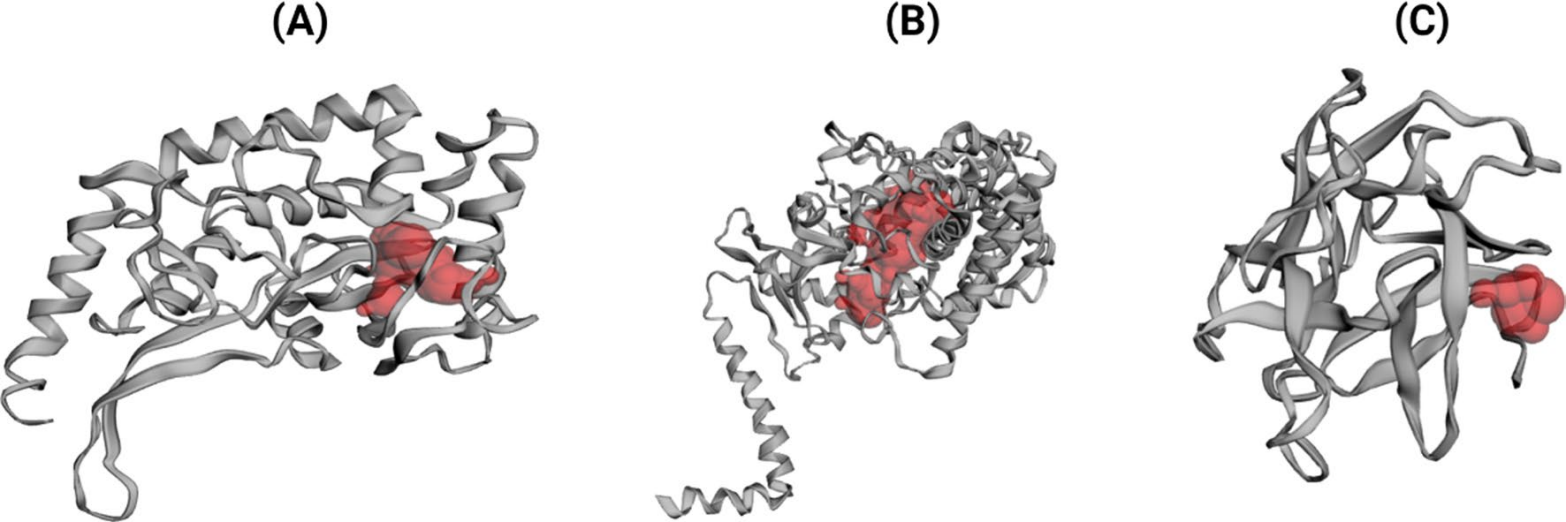
Predicted ligand-binding pockets of CotH3 (**a**), lanosterol 14 alpha-demethylase (**b**), mucoricin (**c**) via CASTp server

CASTp server (Tian et al. 2018) revealed 57, 207 and 17 active sites for CotH3, Lanosterol 14 alpha-demethylase and Mucoricin, respectively. The best pockets showed an area and volume of 191.888 (SA) and 70.401 (SA) for CotH3; 1007.880 (SA) and 653.358 (SA) for Lanosterol 14-alpha demethylase; 38.816 (SA) and 9.453 (SA) for Mucoricin protein (Fig. 4).

**Fig. 5:**
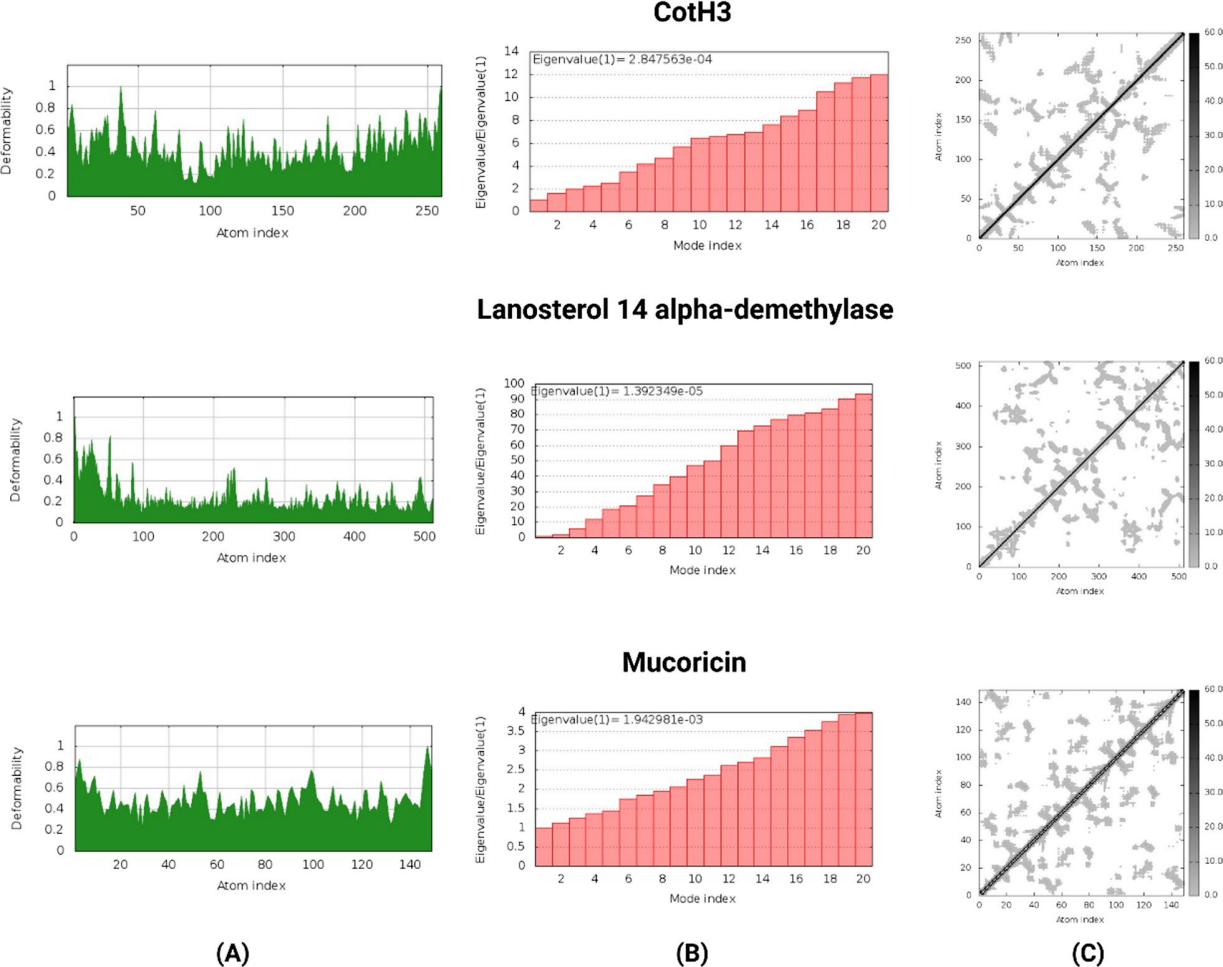
Deformability (**a**), eigen value (**b**) and elastic network (**c**) of *Rhizopus delemar* proteins CotH3, Lanosterol 14 alpha-demethylase and Mucoricin

The deformability, eigenvalue and elastic network of the modelled structures were used to determine their stability. The main chain deformability of the *Rhizopus delemar* proteins are a measure of the capability of a given molecule to deform at each of its residues. The chain 'hinges' location can be derived from high deformability regions (Fig. 5). The higher eigenvalues of CotH3 (2.847563e−04), Lanosterol 14 alpha-demethylase (1.392349e−05) and Mucoricin (1.942981e−03) are representatives of higher energy which is required to deform the protein structures (Fig. 5). As shown in Fig. 5, the elastic network models defined the pairs of atoms connected by springs, where dots are coloured according to the degree of stiffness.

### Binding interactions of ligands With *Rhizopus delemar* CotH3

**Fig. 6:**
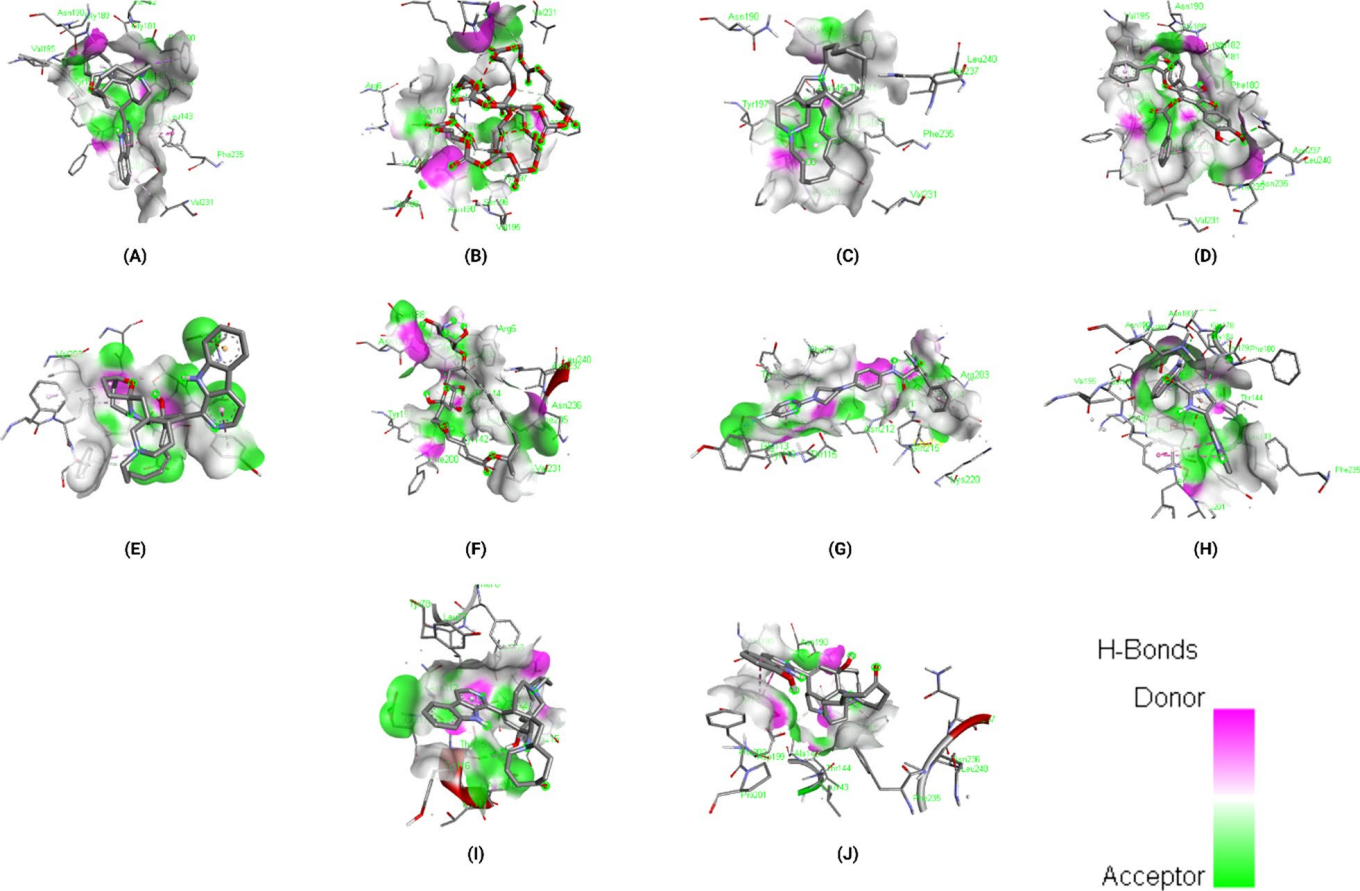
3D visualisation of docking analysis of CotH3 binding with 12,28-oxamanzamine A (**a**), parsiguine (**b**), haliclonacyclamine B (**c**), vialinin B (**d**), 6-deoxymanzamine X (**e**), natamycin (**f**), olorofim (**g**), deoxytopsentin (**h**), manzamine E (**i**), fascioquinol A (**j**)

The binding energies of the selected ligands with the modelled CotH3 were studied. The docking results are given regarding the binding affinity, bond categories, bond length and interacting amino acid residues present at the protein's binding pocket (Table 4). The top 10 ligands are mentioned in Table 4, namely, 12,28-Oxamanzamine A, Parsiguine, Haliclonacyclamine B, Vialinin B, 6-Deoxymanzamine X, Natamycin, Olorofim, Deoxytopsentin, Manzamine E and Fascioquinol A with binding affinities ranging from −8.2 to −10.2 kcal/mol. Of the top 10 lead compounds, 12,28-Oxamanzamine A displayed the best binding affinity (−10.2 kcal/mol) with the *Rhizopus delemar* CotH3. The detailed interaction analysis data of the Top 10 ligands are also provided in Table 4. Further, 3D structural views and 2D depiction of the ligand-binding site interactions are provided in Fig. 6 and Additional file 2: Fig. S1.

**Table 4:**
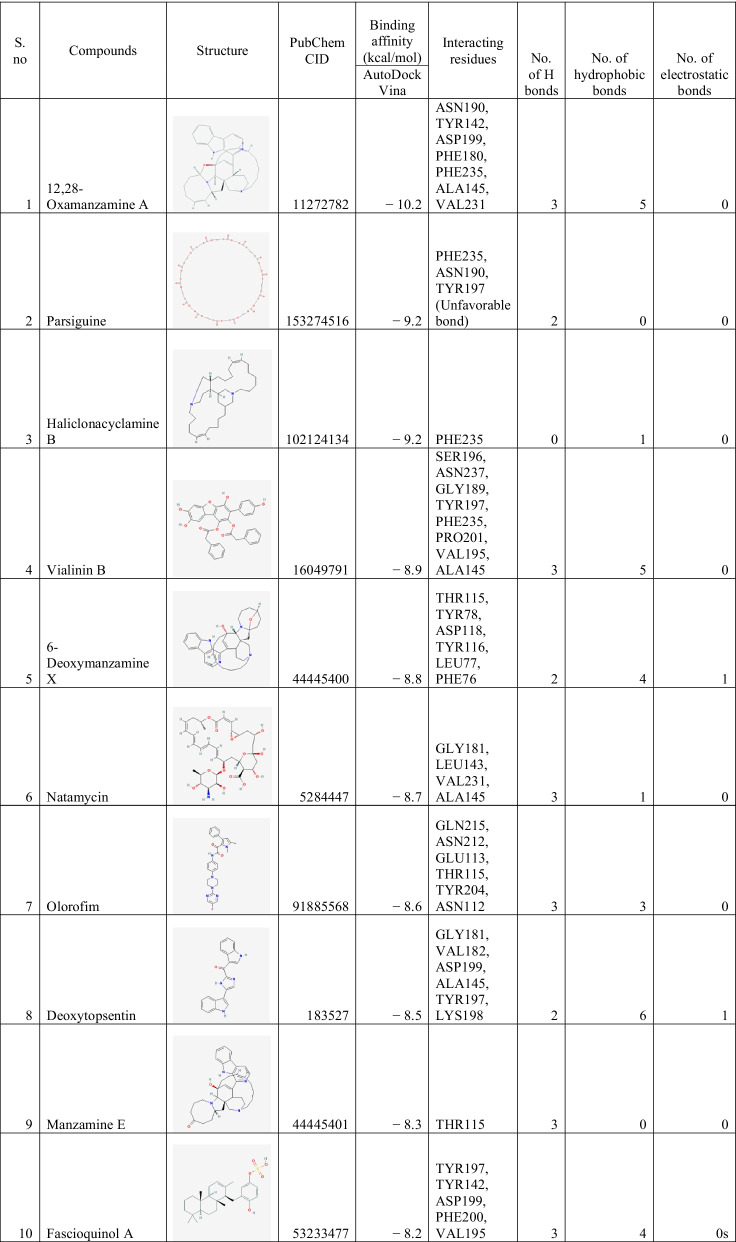
The binding affinity and interaction pattern analysis of top 10 ligands docked with CotH3

### Binding interactions of ligands with *Rhizopus delemar* Lanosterol 14 alpha-demethylase

**Fig. 7:**
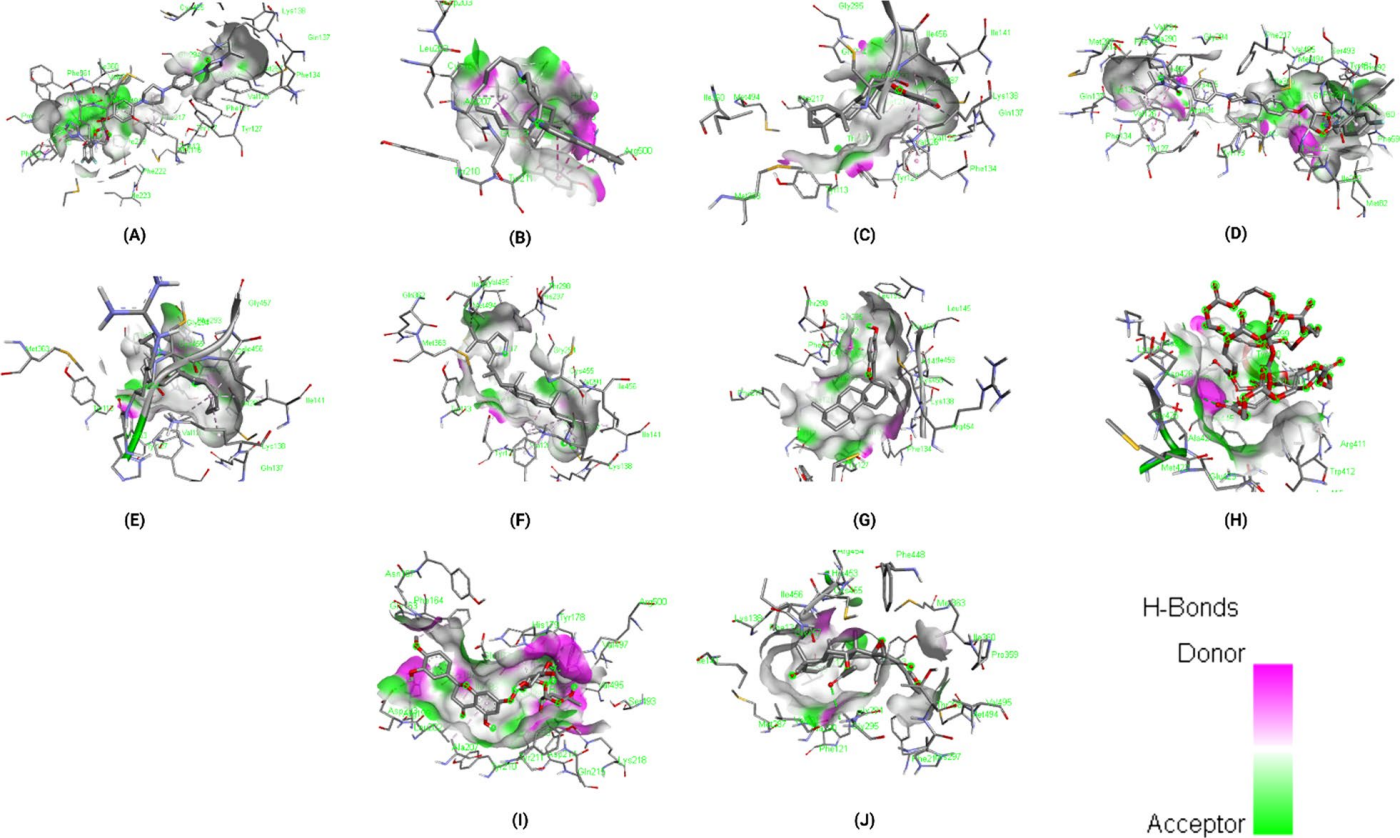
3D visualisation of docking analysis of Lanosterol 14 alpha-demethylase binding with pramiconazole (**a**), 12,28-oxamanzamine A (**b**), fascioquinol D (**c**), saperconazole (**d**), nakadomarin A (**e**), plakinamine A (**f**), fascioquinol C (**g**), parsiguine (**h**), hesperidin (**i**), epoxyazadiradione (**j**)

The docking results of the top 10 ligands with *Rhizopus delemar* Lanosterol 14 alpha-demethylase are provided in Table 5; binding affinities range from −9.9 to −11 kcal/mol. Pramiconazole showed the highest binding affinity (-11 kcal/mol) with Lanosterol 14 alpha-demethylase. The detailed interaction analysis data of the Top 10 ligands are also provided in Table 5. Further, 3D structural views and 2D depiction of the ligand-binding site interactions are provided in Fig. 7 and Additional file 2: Fig. S2.

**Table 5:**
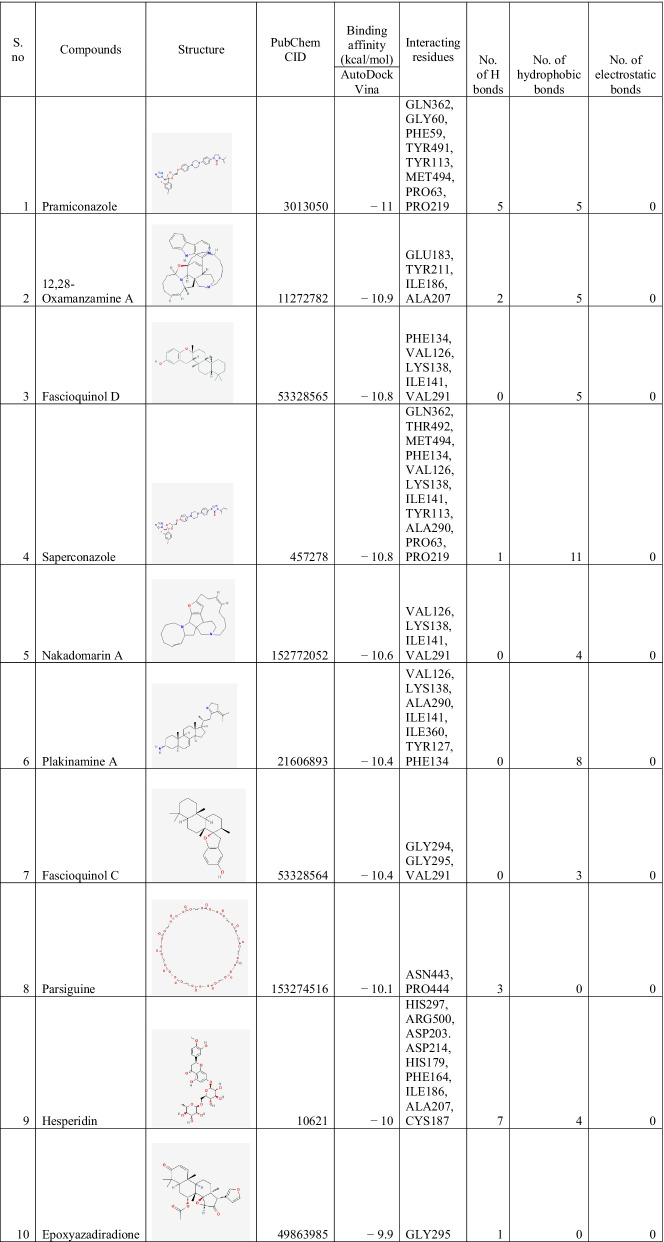
The binding affinity and interaction pattern analysis of top 10 ligands docked with Lanosterol 14 alpha-demethylase

### Binding interactions of ligands With *Rhizopus delemar* Mucoricin

**Fig. 8:**
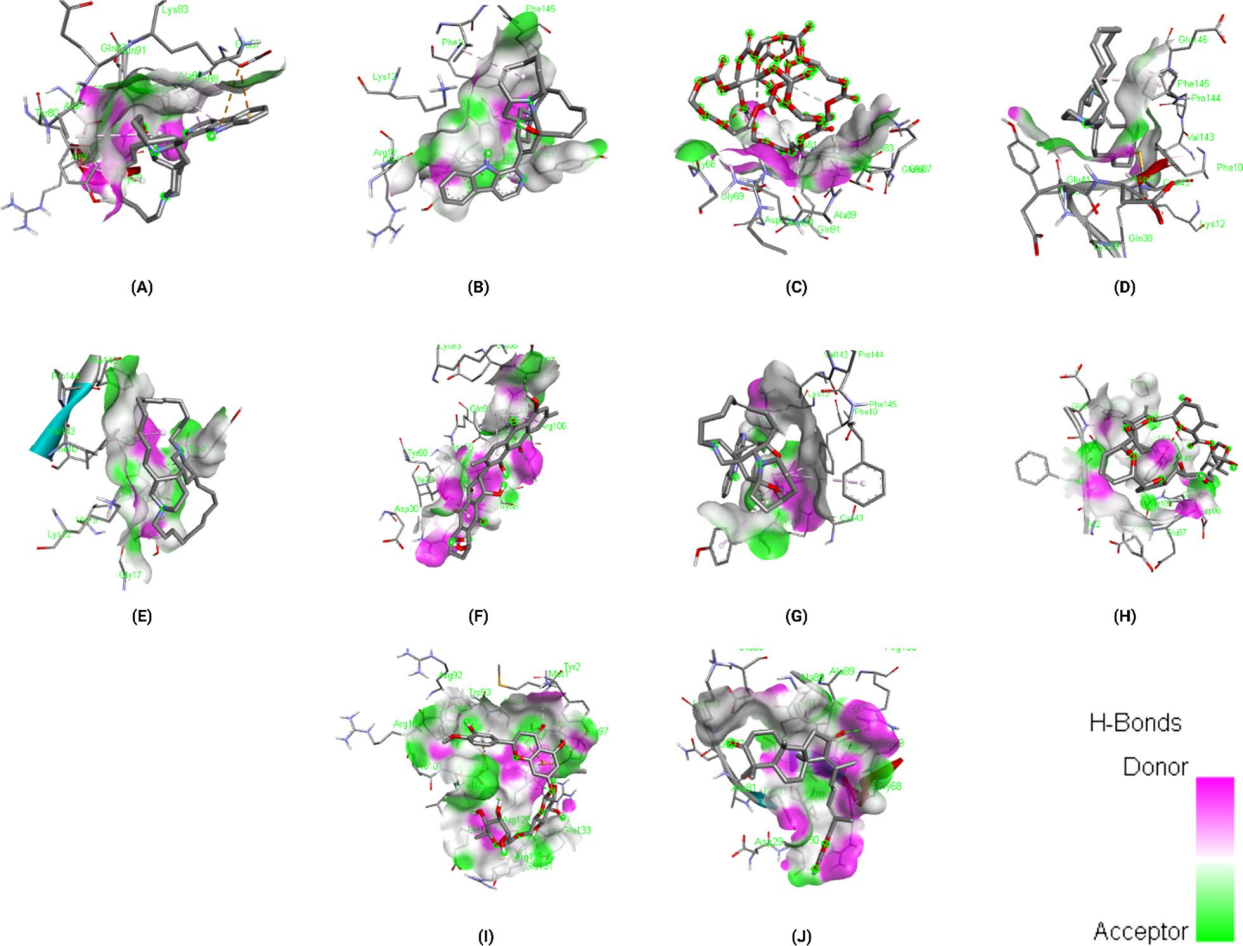
3D visualisation of docking analysis of Mucoricin binding with 12,28-oxamanzamine A (**a**), manzamine A (**b**), parsiguine (**c**), halicyclamine A (**d**), tetrahydrohaliclonacyclamine A (**e**), Phaeosphenone (**f**), 6-deoxymanzamine X (**g**), goniodomin A (**h**), hesperidin (**i**), stelletin A (**j**)

The binding affinities of the top 10 ligands with *Rhizopus delemar* Mucoricin are provided in Table 6; binding affinities were ranged from −7.8 to −8.6 kcal/mol. 12,28-Oxamanzamine A showed the highest binding affinity (−8.6 kcal/mol) with Mucoricin. The detailed interaction analysis data of the Top 10 ligands are also provided in Table 6. Further, 3D structural views and 2D depiction of the ligand-binding site interactions are provided in Fig. 8 and Additional file 2: Fig. S3.

**Table 6:**
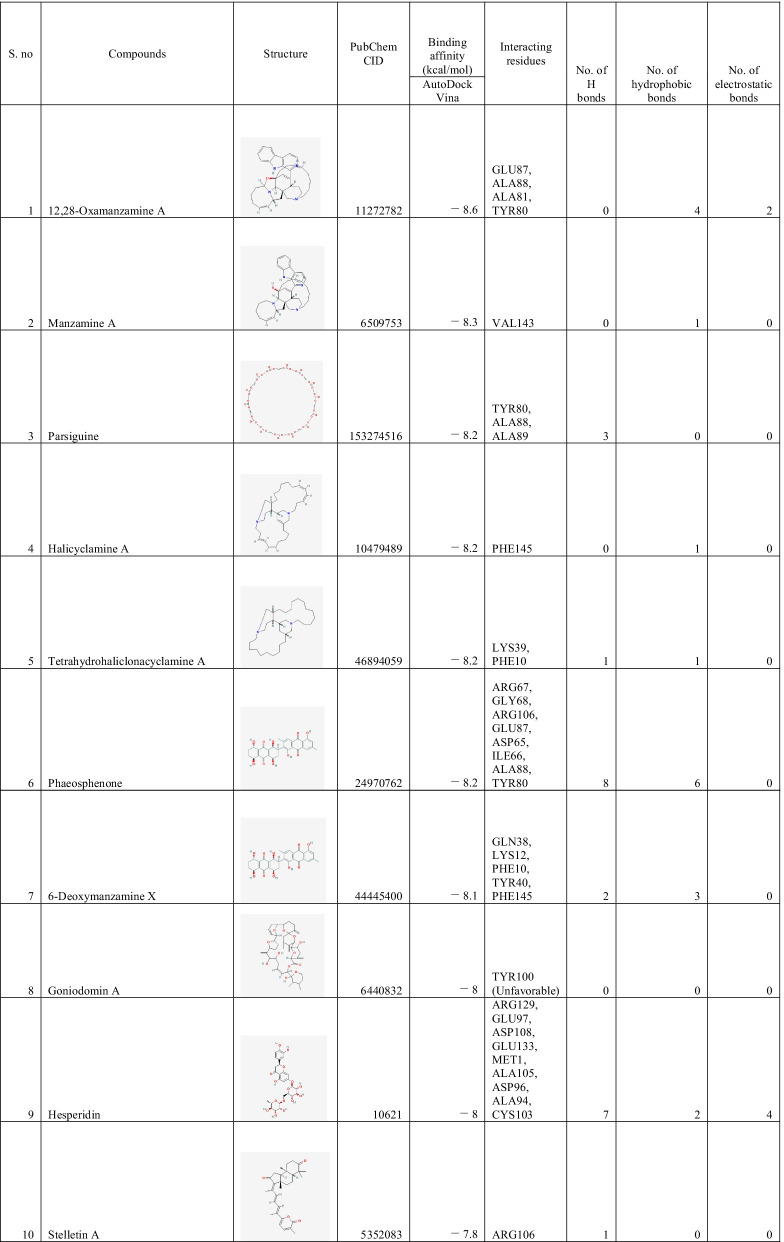
The binding affinity and interaction pattern analysis of top 10 ligands docked with Mucoricin

### Drug profile analysis of top lead compounds, toxicity pattern analysis and in silico bioactivity prediction

Several ADME features of top ligands, including physicochemical parameters, lipophilicity, water-solubility, pharmacokinetics, drug-likeness and medicinal chemistry, are presented to assess their druggability potential (Table 7). The oral bioavailability of the possible active compounds was calculated through Lipinski's rule of five and Veber's rule, while Muegge's rule determined the possibility of a compound to become a successful drug molecule by the pharmacophore point calculation (Muegge et al. 2001). But several drugs do not always follow the drug-likeness rule. There are undoubtedly many notable examples of successful drugs that violate at least two of Lipinski's rules: HMG-CoA reductase inhibitor atorvastatin and leukotriene receptor antagonist montelukast (Beyond the Rule of Five 2021). In a study conducted in 2021(Protti et al. 2021), researchers stated that the selection of drug-like compounds is no longer driven by fixed parameters but by a balance between their physicochemical properties.

**Table 7:** ADME analysis of top ligands docked against our 3 target proteins

S.no	Compounds	SwissADME						
		Lipophilicity	Water Solubility	Pharmacokinetics	Druglikeness			Medicinal Chemistry
		Consensus Log Po/w	Class	GI absorption	Lipinski violations	Veber violations	Muegge violations	Bioavailability Score
1	12,28-Oxamanzamine A	5.3	Poorly soluble	High	2	0	2	0.17
2	Parsiguine	0.74	Soluble	Low	2	1	3	0.17
3	Haliclonacyclamine B	6.58	Moderately soluble	Low	1	0	1	0.55
4	Vialinin B	5.08	Insoluble	Low	1	1	1	0.55
5	6-Deoxymanzamine X	4.7	Poorly soluble	High	1	0	2	0.55
6	Natamycin	− 0.49	Soluble	Low	3	1	4	0.17
7	Olorofim	3.45	Poorly soluble	High	0	0	0	0.55
8	Deoxytopsentin	3.24	Poorly soluble	High	0	0	0	0.55
9	Manzamine E	4.42	Poorly soluble	High	1	0	1	0.55
10	Fascioquinol A	5.33	Poorly soluble	Low	1	0	1	0.56
11	Pramiconazole	4.06	Poorly soluble	High	2	0	1	0.17
12	Fascioquinol D	5.99	Poorly soluble	Low	1	0	1	0.55
13	Saperconazole	4.28	Poorly soluble	High	2	1	1	0.17
14	Nakadomarin A	4.16	Moderately soluble	High	0	0	0	0.55
15	Plakinamine A	5.99	Poorly soluble	High	1	0	1	0.55
16	Fascioquinol C	6.02	Poorly soluble	Low	1	0	1	0.55
17	Hesperidin	− 0.72	Soluble	Low	3	1	4	0.17
18	Epoxyazadiradione	3.93	Poorly soluble	High	0	0	0	0.55
19	Manzamine A	4.98	Poorly soluble	High	2	0	2	0.17
20	Halicyclamine A	6.13	Moderately soluble	Low	1	0	1	0.55
21	Tetrahydrohaliclonacyclamine A	7.26	Poorly soluble	Low	1	0	1	0.55
22	Phaeosphenone	1.31	Moderately soluble	Low	2	1	2	0.17
23	Goniodomin A	3.29	Soluble	Low	2	1	3	0.17
24	Stelletin A	5.97	Poorly soluble	High	1	0	1	0.55

The ligands were then tested for toxicity using an online tool called ProTox-II and StopTox, machine learning tools (Banerjee et al. 2018; Borba et al. 2020). In ProTox-II, there are six toxicity classes (1–6) based on a globally harmonised system of classification of labelling of chemicals (GHS). LD50 values are given in mg/kg and the classes are described as: Class I: death if swallowed (LD50 ≤ 5); Class 2: fatal if swallowed (5 < LD50 ≤ 50); Class 3: toxic if swallowed (50 < LD50 ≤ 300); Class 4: harmful if swallowed (300 < LD50 ≤ 2000); Class 5: may be harmful if swallowed (2000 < LD50 ≤ 5000); Class 6: non-toxic (LD50 > 5000) (Abel et al. 2020). LD50 stands for Lethal Dose 50 which is a measure the amount of a substance needed to kill half of a test population of animals (What is LD50 2021). This study demonstrated how likely and successful a medicine might be with a minimal number of adverse effects and provided us with a prediction score. On the other hand, StopTox was used for assessing the potential of chemicals to cause acute toxicity. Toxicity predicted by ProTox‑II and StopTox is summarised in Table 8.

**Table 8:** Toxicity report carried out using ProTox-II and STopTox server for top ligand compounds

S. no	Ligand name	ProTox-II	STopTox				
		predicted LD50 (mg/kg)	Toxicity class	Acute inhalation Toxicity	Acute dermal toxicity	Eye irritation and corrosion	Skin sensitisation
1	12,28-Oxamanzamine A	4	1	Non-toxic (−)	Non-toxic (−)	Toxic (+)	Non-sensitizer (−)
2	Parsiguine	850	4	Non-toxic (−)	Non-toxic (−)	Toxic (+)	Sensitizer (+)
3	Haliclonacyclamine B	652	4	Toxic (+)	Toxic (+)	Toxic (+)	Sensitizer (+)
4	Vialinin B	5000	5	Non-toxic (−)	Toxic (+)	Non-toxic (−)	Sensitizer (+)
5	6-Deoxymanzamine X	4	1	Non-toxic (−)	Non-toxic (−)	Toxic (+)	Non-sensitizer (−)
6	Natamycin	1500	4	Non-toxic (−)	Non-toxic (−)	Toxic (+)	Non-sensitizer (−)
7	Olorofim	1420	4	Non-toxic (−)	Non-toxic (−)	Toxic (+)	Non-sensitizer (−)
8	Deoxytopsentin	1264	4	Non-toxic (−)	Non-toxic (−)	Non-toxic (−)	Non-sensitizer (−)
9	Manzamine E	9	2	Non-toxic (−)	Non-toxic (−)	Toxic (+)	Non-sensitizer (−)
10	Fascioquinol A	2000	4	Toxic (+)	Non-toxic (−)	Non-toxic (−)	Non-sensitizer (−)
11	Pramiconazole	320	4	Non-toxic (−)	Non-toxic (−)	Toxic (+)	Non-sensitizer (−)
12	Fascioquinol D	5000	5	Non-toxic (−)	Non-toxic (−)	Non-toxic (−)	Sensitizer (+)
13	Saperconazole	4000	5	Non-toxic (−)	Non-toxic (−)	Toxic (+)	Non-sensitizer (−)
14	Nakadomarin A	1000	4	Toxic (+)	Non-toxic (−)	Toxic (+)	Non-sensitizer (−)
15	Plakinamine A	1000	4	Toxic (+)	Toxic (+)	Non-toxic (−)	Non-sensitizer (−)
16	Fascioquinol C	1743	4	Non-toxic (−)	Non-toxic (−)	Non-toxic (−)	Sensitizer (+)
17	Hesperidin	12,000	6	Non-toxic (−)	Toxic (+)	Non-toxic (−)	Non- Sensitizer (−)
18	Epoxyazadiradione	555	5	Non-toxic (−)	Non-toxic (−)	Non-toxic (−)	Non- Sensitizer (−)
19	Manzamine A	4	1	Non-toxic (−)	Non-toxic (−)	Toxic (+)	Non-sensitizer (−)
20	Halicyclamine A	460	4	Toxic (+)	Toxic (+)	Toxic (+)	Sensitizer (+)
21	Tetrahydrohaliclonacyclamine A	194	3	Toxic (+)	Toxic (+)	Toxic (+)	Sensitizer (+)
22	Phaeosphenone	221	3	Non-toxic (−)	Non-toxic (−)	Non-toxic (−)	Non-sensitizer (−)
23	Goniodomin A	500	4	Non-toxic (−)	Non-toxic (−)	Non-toxic (−)	Non-sensitizer (−)
24	Stelletin A	800	4	Non-toxic (−)	Non-toxic (−)	Non-toxic (−)	Sensitizer (+)

Analysis of the structure–activity relationship for a complete training set involving drug compounds, drug candidates in numerous clinical and preclinical study steps, and pharmaceutical agents are the basis of prediction in the PASS program (Lagunin et al. 2000). The mechanisms of action and pharmacological activities, calculated probabilities for the exhibition of activity exceeding the probability verge (Pa > Pi), existed in the default list of predicted effects. The Pa and Pi values vary in the range of 0.000–1.000, and, in general, the summation of Pa and Pi should not equal one. For a compound, the chance to achieve the desired experimental activity is high when Pa > 0.7. Suppose a compound is likely to exhibit the activity in the experiment. In that case, the chance to find the experimental activity will be less, and the compound is probably not so similar to a known pharmaceutical agent (0.5 < Pa < 0.7). A compound is unlikely to display the activity recognised in the experiment when Pa < 0.5, and this compound might be a new chemical entity. The top compounds which have satisfactory ADME and toxicity properties are subjected to bioactivity prediction in which activities that have Pa ≥ 0.7 is selected and summarised in Table 9. The bioactivity scores (ion channel modulation (ICM), G protein-coupled receptor (GPCR), nuclear receptor ligand (NRL) and enzyme inhibitors: protease, kinase) of the top ligands were predicted by using Molinspiration Cheminformatics online server (Table 9).

**Table 9:** Bioactivity prediction report of filtered top compounds

S. no	Ligands	Target	Passonline	Molinspiration					
			biology activity (Pa > 0.7)	GPCR ligand	Ion channel modulator	Kinase inhibitor	Nuclear receptor ligand	Protease inhibitor	Enzyme inhibitor
1	12,28-Oxamanzamine A	CotH3, Lanosterol 14 alpha-demethylase, Mucoricin	Antineoplastic alkaloid	0.38	− 0.09	0.36	− 0.04	0.17	0.07
2	Haliclonacyclamine B	CotH3	Cognition disorders treatment, Antipsychotic,	0.24	0.16	− 0.01	0.06	0.15	0.11
3	Vialinin B	CotH3	Histidine kinase inhibitor, Chlordecone reductase inhibitor, HIF1A expression inhibitor	0.03	− 0.48	− 0.25	0.08	0.03	− 0.1
4	Olorofim	CotH3	Nil	0.109	0.218	0.227	− 0.269	− 0.26	− 0.006
5	Deoxytopsentin	CotH3	Antineoplastic alkaloid	0.468	0.119	0.635	0.013	− 0.2	0.418
6	Pramiconazole	Lanosterol 14 alpha-demethylase	Antifungal	− 0.2	− 1.06	− 0.74	− 1.01	− 0.15	− 0.69
7	Fascioquinol D	Lanosterol 14 alpha-demethylase	Oxidoreductase inhibitor, Chemopreventive, Antineoplastic	0.21	0.25	− 0.15	0.59	0.08	0.37
8	Saperconazole	Lanosterol 14 alpha-demethylase	Lanosterol 14 alpha demethylase inhibitor, Antifungal, CYP51 inhibitor	− 0.41	− 1.5	− 1.24	− 1.27	− 0.61	− 0.96
9	Fascioquinol C	Lanosterol 14 alpha-demethylase	Oxidoreductase inhibitor, Phosphatase inhibitor, Hypolipemic	0.05	0.2	− 0.32	0.49	− 0.12	0.3
10	Parsiguine	Mucoricin	Antineoplastic (lung cancer), Antieczematic atopic, Sugar-phosphatase inhibitor, Glycosylphosphatidylinositol phospholipase D inhibitor	− 3.75	− 3.83	− 3.83	− 3.84	− 3.69	− 3.79
11	Halicyclamine A	Mucoricin	Analgesic, non-opioid, Cognition disorders treatment	0.35	0.33	0.04	0.08	0.15	0.21
12	Tetrahydrohaliclonacyclamine A	Mucoricin	Antipsychotic, Cognition disorders treatment, Cardiovascular analeptic	0.2	0.15	− 0.01	0.03	0.15	0.06
13	Hesperidin	Mucoricin	Free radical scavenger, Beta glucuronidase inhibitor, Alpha glucosidase inhibitor, UDP-glucuronosyltransferase substrate	− 0.01	− 0.59	− 0.36	− 0.2	0	0.06

## Discussion

According to recent observations, individuals who are in highly immune-compromised health circumstances following COVID-19 having diabetes or high uncontrolled sugar levels were infected with a disease produced by a "mucormycosis" (Sharma and Kaur 2021). The two agents currently approved by the FDA for the primary treatment of mucormycosis are amphotericin B and isavuconazole (Bhattacharya and Setia 2021). Previous research efforts to develop antifungal agents against the Mucorales demonstrated that the inhibition of β-1,3-glucan biosynthesis by using inhibitor drugs like amphotericin/echinocandins inhibited fungal growth, thus abolished replication (Sharma and Kaur 2021). In the study conducted in 2014, researchers suggested that CotH3 could be an emerging therapeutic target for mucormycosis as this functions as an invasin that interacts with host cell GRP78 to mediate pathogenic host-cell interactions (Gebremariam et al. 2014). Similarly, inhibition of Lanosterol 14 alpha-demethylase interrupts the conversion of lanosterol to ergosterol, which leads to the depletion of ergosterol in the fungal cell membrane and accumulation of aberrant 14-α-methylsterols in fungal cells, thereby causing fungal death (Shoham et al. 2017). Further, they produce a toxin called mucoricin, which plays a central role in the virulence of Mucorales (Soliman et al. 2021). Hence, targeting CotH3, Lanosterol 14 alpha-demethylase and Mucoricin may offer a new active antifungal approach to treat mucormycosis. Thus, in our study, we attempted to reveal a novel therapeutic option for treating mucormycosis by the screening of FDA approved drugs, FDA unapproved, investigational-only, natural compounds against our targeted proteins using structure-based virtual screening. To date, no crystal structures were determined for our *Rhizopus delemar* target proteins. Hence, protein modelling was performed for the prediction of protein structure based on the available sequence data. The 3D-modelled structures were thoroughly investigated and confirmed using the Ramachandran Plot analysis. Moreover, ascertainment of stability can be done by comparing proteins essential dynamics to their normal modes. The protein models were stable and showed some deformability at the molecular level (Additional file 3).

Approaches such as virtual screening and de novo drug creation are powerful tools for identifying lead compounds with targeted biological activity. Analysing the interactions of macromolecules and small ligands is an efficient approach to simplify the path of current drug discovery while also reducing the time and expense of the drug development process. Molecular docking using AutoDock Vina results showed that 12,28-Oxamanzamine A, Parsiguine, Haliclonacyclamine B, Vialinin B, 6-Deoxymanzamine X, Natamycin, Olorofim, Deoxytopsentin, Manzamine E and Fascioquinol A were the top leads for CotH3; Pramiconazole, 12,28-Oxamanzamine A, Fascioquinol D, Saperconazole, Nakadomarin A, Plakinamine A, Fascioquinol C, Parsiguine, Hesperidin and Epoxyazadiradione were the top leads for Lanosterol 14 alpha-demethylase; 12,28-Oxamanzamine A, Manzamine A, Parsiguine, Halicyclamine A, Tetrahydrohaliclonacyclamine A, Phaeosphenone 6-Deoxymanzamine X, Goniodomin A, Hesperidin and Stelletin A were the top leads for Mucoricin; and the top leads regarding minimum global binding energy (Tables 4, 5, 6). Notably, 12,28-Oxamanzamine A was seen in all three proteins as a lead compound. Further ADME profiling and toxicity analysis were performed to investigate how our lead compounds are processed by a living organism (Tables 7, 8). It revealed that most of the lead compounds are highly toxic in nature and possess satisfactory ADME properties. The 12,28-Oxamanzamine and five other compounds were further filtered using these properties for each protein and subjected to bioactivity prediction (Pa > 0.7) (Table 9). In addition, we compared the binding affinities of currently prescribed mucormycosis drugs to our shortlisted candidates for the three target proteins (Table 10). Posaconazole and isavuconazole had a high affinity for Lanosterol 14 alpha-demethylase, but not for other protein targets. However, there are also other selected compounds such 12,28-Oxamanzamine A, pramiconazole, and saperconazole that exhibited a higher affinity for Lanosterol 14 alpha-demethylase than posaconazole and isavuconazole. Overall, our shortlisted compounds have good binding affinities with all three protein targets than the currently prescribed drugs.

**Table 10:** Comparison of docking results between currently prescribed drugs and selected bioactive compounds against three target proteins

	Compounds	Binding affinity (kcal/mol) with CotH3	Binding affinity (kcal/mol) with Lanosterol 14 alpha-demethylase	Binding affinity (kcal/mol) with mucoricin
Selected candidates	12,28-Oxamanzamine A	− 10.2	− 10.9	− 8.6
	Vialinin B	− 8.9	− 7.8	− 6.5
	Deoxytopsentin	− 8.5	− 9.5	− 7.2
	Pramiconazole	− 7.6	− 11	− 7.1
	Saperconazole	− 7.8	− 10.8	− 7
	Hesperidin	− 8	− 10	− 8
Currently prescribed drugs for mucormycosis	Posaconazole	− 7.8	− 9.8	− 6.4
	Isavuconazole	− 6.5	− 9.2	− 5.9

The detailed elucidation on the molecular properties and the interaction profiles of the shortlisted six bioactive compounds against *Rhizopus delemar* proteins are as follows:

### 28-Oxamanzamine A

It is isolated from a common Indonesian sponge of the genus Acanthostrongylophora. It has potent anti-inflammatory, antifungal and anti-HIV-1 activity (Yousaf et al. 2004). It showed a high binding affinity with all our three targeted proteins, CotH3 (−10.2 kcal/mol), Lanosterol 14 alpha-demethylase (− 10.9 kcal/mol) and Mucoricin (− 8.6 kcal/mol). The molecule has a molecular weight of 546.7 g/mol, 4 H-bond acceptors and 1 H-bond donor, formed three H-bonds with ASN190, TYR142 and ASP199 amino acid residues and five Hydrophobic bonds with PHE180, PHE235, PHE235, ALA145 and VAL231 amino acid residues of CotH3. In contrast, for Lanosterol 14 alpha-demethylase it formed two H-bonds with GLU183 amino acid residues and five hydrophobic interactions with TYR211, ILE186 ALA207 amino acid residues. Similarly, for Mucoricin it formed two electrostatic bonds with GLU87 amino acid residue and four hydrophobic interactions with ALA88, ALA81 and TYR80. ADME analysis revealed that this molecule has poor water solubility and consensus Log Po/w value of 5.3 with high GI absorption while having a poor bioavailability score of 0.17. Toxicity results showed that this molecule was toxic with the predicted LD50 of 4 mg/kg. Although it was fatal, its bioactivity score by molinsipiration revealed that it could probably act as a suitable kinase inhibitor (0.36) and an antineoplastic alkaloid. Since CotH3 is a protein kinase, there might be a chance that 12,28-Oxamanzamine A could act as a CotH3 inhibitor. Also, it displayed a high binding affinity for all our target proteins. So, further ADME and toxicity optimisation are needed to evaluate its performance in vitro and in vivo studies.

### Vialinin B

It is a novel dibenzofuran compound isolated from dry fruiting bodies of an edible mushroom, Thelephora vialis, which potently inhibits TNF-alpha production in RBL-2H3 cells (IC (50) = 0.02 nM) and acts as a promising anti-allergic agent (X. C et al. 2006). It displayed a binding affinity of −8.9 kcal/mol with CotH3. The molecule has a molecular weight of 576.5 g/mol, 9 H-bond acceptors and 4 H-bond donors, formed 3 H-bonds with SER196, ASN237 and GLY189 amino acid residues and five hydrophobic interactions with TYR197, PHE235, PRO201, VAL195 and ALA145. ADME analysis revealed that this molecule is insoluble in water with the consensus Log Po/w value of 5.08 with low GI absorption and a good bioavailability score of 0.55. Toxicity results showed that this molecule was less toxic with the predicted LD50 of 5000 mg/kg. Bioactivity prediction revealed that this molecule could act as a Histidine kinase inhibitor, Chlordecone reductase inhibitor and HIF1A expression inhibitor. Since it can act as a kinase inhibitor, it might be a possibility to inhibit the CotH3 protein.

### Deoxytopsentin

It is a naturally occurring sponge metabolite that acts as a bisindole alkaloid inhibitor against the evolutionary conserved MRSA pyruvate kinase (PK). The compound displayed potent low nanomolar inhibitory activity against MRSA PK with significant concomitant selectivity over human PK orthologues (Veale et al. 2015). It showed a binding affinity of −8.5 kcal/mol with CotH3. The molecule has a molecular weight of 326.4 g/mol, 2 H-bond acceptors and 3 H-bond donors, formed 2 H-bonds with GLY181 and VAL182 amino acid residues, one electrostatic bond with ASP199 and six hydrophobic interactions with ASP199, TYR197, ALA145 and LYS198 amino acid residues. ADME analysis revealed that this molecule was poorly soluble in water with the consensus Log Po/w value of 3.24 with high GI absorption and a good bioavailability score of 0.55. Toxicity results showed that this molecule has a toxicity class of 4 with the predicted LD50 of 1264 mg/kg. Its bioactivity score by molinsipiration revealed that it could probably act as a suitable kinase inhibitor (0.635) and an antineoplastic alkaloid. So, it might act as an antagonist for CotH3.

### Pramiconazole

Pramiconazole from Barrier Therapeutics Inc is a new addition to the triazole antifungal agents that inhibit fungal cell membrane ergosterol synthesis, thereby leading to increased cell permeability and destruction. In preclinical studies, pramiconazole exhibited similar or superior antifungal activity to ketoconazole and itraconazole and selectively inhibited ergosterol synthesis with a broad-spectrum activity (Wit et al. 2010). It showed a binding affinity of −11.0 kcal/mol with Lanosterol 14 alpha-demethylase. The molecule has a molecular weight of 659.7 g/mol, 8 H-bond acceptors and 0 H-bond donors, formed five H-bonds with GLN362, GLY60, PHE59, GLY60 and TYR491 amino acid residues and five hydrophobic interactions with PHE59, TYR113, MET494, PRO63 and PRO219 amino acid residues. ADME analysis revealed that this molecule is poorly soluble in water with the consensus Log Po/w value of 4.06 with high GI absorption and a poor bioavailability score of 0.17. Toxicity results showed that this molecule has a toxicity class of 4 with the predicted LD50 of 320 mg/kg. Its bioactivity prediction revealed that this compound is antifungal, and it was experimentally verified by another study to inhibit the lanosterol 14 alpha-demethylase (Wit et al. 2010).

### Saperconazole

The N-1-substituted triazole antifungal, saperconazole, is a potent inhibitor of ergosterol synthesis in Candida albicans, Aspergillus fumigatus and Trichophyton mentagrophytes. Fifty % inhibition is already achieved at nanomolar concentrations. The saperconazole induced inhibition of ergosterol synthesis coincides with an accumulation of 14-methylated sterols, such as 24-methylene-dihydro lanosterol, lanosterol, obtusifoliol, 14α-methylfecosterol, 14α-methylergosta-8,24(28)-dien-3, β-6α-diol and 14α-methylergosta-5,7,22,24(28)-tetraenol (Vanden Bossche et al. 1990). It showed a binding affinity of −10.8 kcal/mol with Lanosterol 14 alpha-methylase. The molecule has a molecular weight of 672.7 g/mol, 9 H-bond acceptors and 0 H-bond donors, formed one H-bond with GLN362 amino acid residue, one halogen bond with THR492 and ten hydrophobic interactions with MET494, PHE134, VAL126, LYS138, ILE141, TYR113, PHE134, VAL126, ALA290, PRO63 and PRO219 amino acid residues. ADME analysis revealed that this molecule was poorly soluble in water with the consensus Log Po/w value of 4.28 with high GI absorption and a poor bioavailability score of 0.17. Toxicity results showed that this molecule has a toxicity class of 5 with the predicted LD50 of 4000 mg/kg, and it was experimentally verified by Bossche H Vanden to inhibit the lanosterol 14 alpha-demethylase (Vanden Bossche et al. 1990).

### Hesperidin

Hesperidin is a flavanone glycoside found in citrus fruits. Its name is derived from "hesperidium", which stands for "fruit from citrus trees". It exhibits various biological properties, including antioxidant, anti-inflammatory and anti-cancer effects. Recent studies indicated that it possesses antimicrobial activity (Iranshahi et al. 2015). It displayed a − 10.0 kcal/mol binding affinity with Lanosterol 14 alpha-demethylase and −8 kcal/mol with Mucoricin. The molecule has a molecular weight of 610.6 g/mol, 15 H-bond acceptors and 8 H-bond donors, formed seven H-bonds with HIS297, ASP203, ASP214, ARG500 and HIS179 amino acid residues and four hydrophobic interactions with PHE164, ILE186, ALA207 and CYS187 amino acid residues of Lanosterol 14 alpha-demethylase. In contrast, for Mucoricin it formed seven H-bonds with ARG129, GLU97, ASP108, GLU133, MET1 and ALA105 amino acid residues, four electrostatic bonds with ARG129, ASP96, GLU97 and ASP108 amino acid residues and two hydrophobic interactions with ALA94 and CYS103 amino acid residues. ADME analysis revealed that this molecule is soluble in water with the consensus Log Po/w value of − 0.72 with low GI absorption and a poor bioavailability score of 0.17. Toxicity results showed that this molecule has a toxicity class of 6 with the predicted LD50 of 12,000 mg/kg. Its bioactivity prediction revealed that it could act as a beta-glucuronidase inhibitor and alpha-glucosidase inhibitor. Since Mucoricin comes under the glycosylases, there could be a high chance that hesperidin can inhibit the Mucoricin protein.

## Conclusions

Mucormycosis emerged as an epidemic in India. In this present study, the possible medications using existing drugs and natural compounds were screened using molecular docking techniques. This research was aimed to identify potent bioactive compounds that could effectively inhibit the potential targets of *Rhizopus delemar*. Our study suggests that 12,28-Oxamanzamine A, vialinin B, deoxytopsentin, pramiconazole, saperconazole and hesperidin could be potent bioactive compounds for the treatment of mucormycosis. Of these, 12,28-Oxamanzamine A has the potential to act as a multi-targeted agent, as it has the highest binding affinity toward the three crucial proteins i.e. CotH3, Lanosterol 14 alpha-demethylase and Mucoricin. However, ADME properties and Toxicity prediction are not favourable for human consumption. So, it needs further ADME and toxicity optimisation to bring out its true potential against mucormycosis. However, the results are solely based on in silico studies. Due to the encouraging results, we highly recommend further in vitro and in vivo trials using animal models for the experimental validation of the findings.

## Supplementary Information

**Additional file 1: Figure S1**. 2D visualisation of docking analysis of CotH3 binding with 12,28-Oxamanzamine A (A), Parsiguine (B), Haliclonacyclamine B (C), Vialinin B (D), 6-Deoxymanzamine X (E), Natamycin (F), Olorofim (G), Deoxytopsentin (H), Manzamine E (I), Fascioquinol A (J). **Figure S2**. 2D visualisation of docking analysis of Lanosterol 14 alpha-demethylase binding with Pramiconazole (A), 12,28-Oxamanzamine A (B), Fascioquinol D (C), Saperconazole (D), Nakadomarin A (E), Plakinamine A (F), Fascioquinol C (G), Parsiguine (H), Hesperidin (I), Epoxyazadiradione (J). **Figure S3**. 2D visualisation of docking analysis of Mucoricin binding with 12,28-Oxamanzamine A (A), Manzamine A (B), Parsiguine (C), Halicyclamine A (D), Tetrahydrohaliclonacyclamine A (E), Phaeosphenone (F), 6-Deoxymanzamine X (G), Goniodomin A (H), Hesperidin (I), Stelletin A (J).

**Additional file 2: Table S1**. The variants of mucormycosis and the symptoms associated with the disease (Kontoyiannis & Lewis, 2011; Ribes et al., 2000; Sheng, 2020; Spellberg et al., 2005; Symptoms of Mucormycosis | Mucormycosis | CDC, n.d.).

**Additional file 3: Data 1**. Binding affinities of all bioactive compunds with our three target proteins.

## Data Availability

All necessary data generated or analyzed during this study are included in this article. Any additional data could be available from the corresponding author upon request.

## Abbreviations

COVID-19: COrona VIrus Disease 2019
SARS-CoV-2: Severe Acute Respiratory Syndrome-Corona Virus-2
FDA: Food and Drug Administration
