# Supplementary material for: In silico prediction and structure-based multitargeted molecular docking analysis of selected bioactive compounds against mucormycosis
Source: Bull Natl Res Cent. 2022 Jan 31;46(1):24. doi: 10.1186/s42269-022-00704-4 (PMC8802264; doi:10.1186/s42269-022-00704-4)
Supplement: Supplementary file 1 — Additional file 1: Figure S1. 2D visualisation of docking analysis of CotH3 binding with 12,28-Oxamanzamine A (A), Parsiguine (B), Haliclonacyclamine B (C), Vialinin B (D), 6-Deoxymanzamine X (E), Natamycin (F), Olorofim (G), Deoxytopsentin (H), Manzamine E (I), Fascioquinol A (J). Figure S2. 2D visualisation of docking analysis of Lanosterol 14 alpha-demethylase binding with Pramiconazole (A), 12,28-Oxamanzamine A (B), Fascioquinol D (C), Saperconazole (D), Nakadomarin A (E), Plakinamine A (F), Fascioquinol C (G), Parsiguine (H), Hesperidin (I), Epoxyazadiradione (J). Figure S3. 2D visualisation of docking analysis of Mucoricin binding with 12,28-Oxamanzamine A (A), Manzamine A (B), Parsiguine (C), Halicyclamine A (D), Tetrahydrohaliclonacyclamine A (E), Phaeosphenone (F), 6-Deoxymanzamine X (G), Goniodomin A (H), Hesperidin (I), Stelletin A (J). [file 42269_2022_704_MOESM1_ESM.docx]

**Supplementary figure legends**

**Supplementary figure 1:** 2D visualization of docking analysis of CotH3 binding with 12,28-Oxamanzamine A (A), Parsiguine (B), Haliclonacyclamine B (C), Vialinin B (D), 6-Deoxymanzamine X (E), Natamycin (F), Olorofim (G), Deoxytopsentin (H), Manzamine E (I), Fascioquinol A (J).

**Supplementary figure 2:** 2D visualization of docking analysis of Lanosterol 14 alpha-demethylase binding with Pramiconazole (A), 12,28-Oxamanzamine A (B), Fascioquinol D (C), Saperconazole (D), Nakadomarin A (E), Plakinamine A (F), Fascioquinol C (G), Parsiguine (H), Hesperidin (I), Epoxyazadiradione (J)

**Supplementary figure 3:** 2D visualization of docking analysis of Mucoricin binding with 12,28-Oxamanzamine A (A), Manzamine A (B), Parsiguine (C), Halicyclamine A (D), Tetrahydrohaliclonacyclamine A (E), Phaeosphenone (F), 6-Deoxymanzamine X (G), Goniodomin A (H), Hesperidin (I), Stelletin A (J).


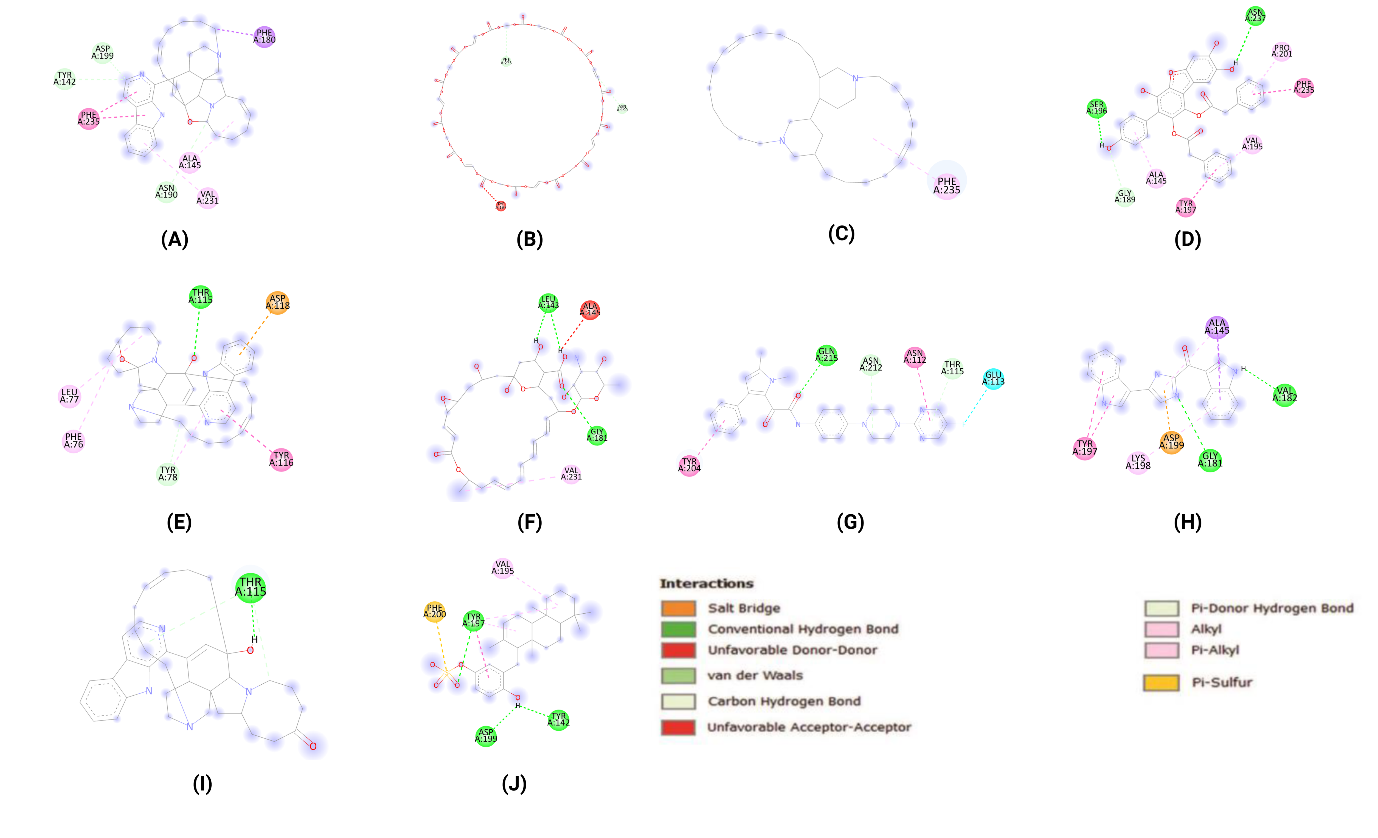


**Supplementary figure 1:** 2D visualization of docking analysis of CotH3 binding with 12,28-Oxamanzamine A (A), Parsiguine (B), Haliclonacyclamine B (C), Vialinin B (D), 6-Deoxymanzamine X (E), Natamycin (F), Olorofim (G), Deoxytopsentin (H), Manzamine E (I), Fascioquinol A (J).


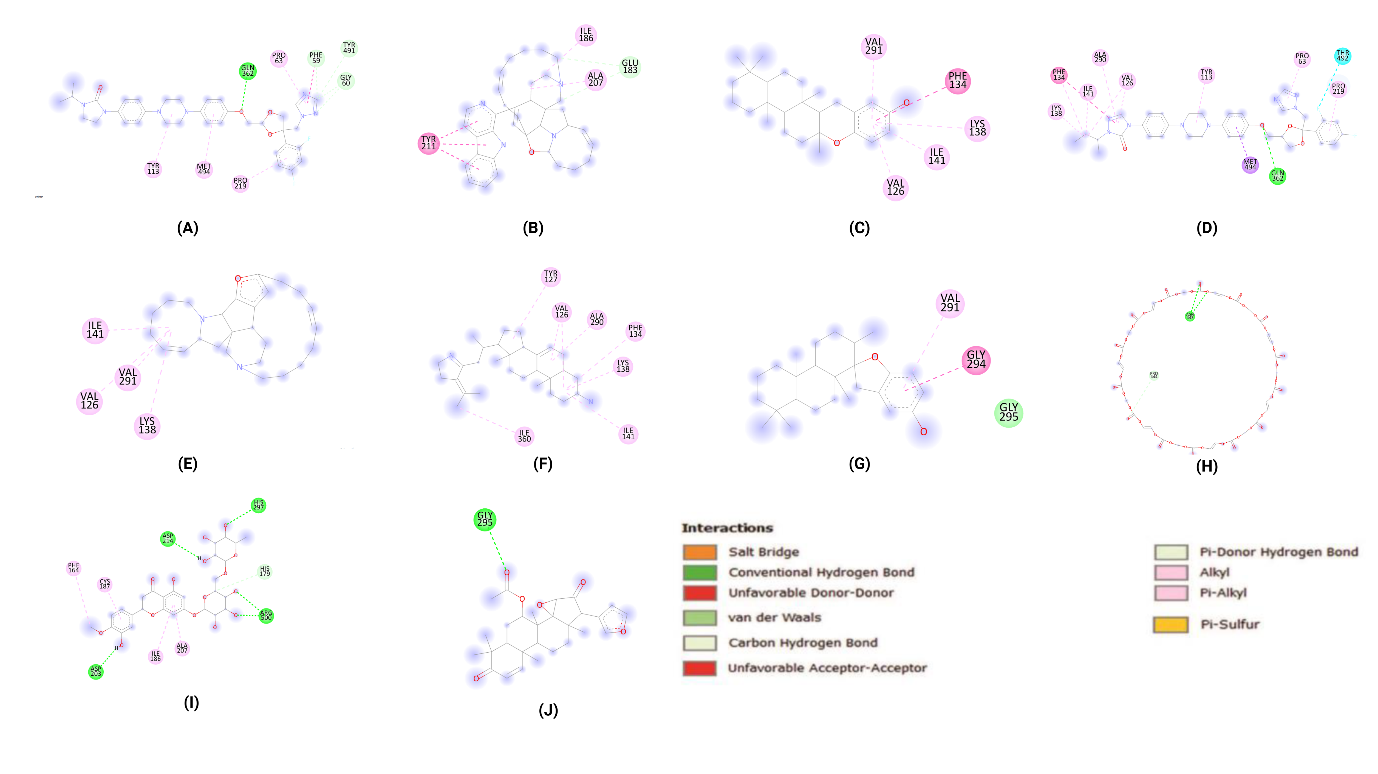


**Supplementary figure 2:** 2D visualization of docking analysis of Lanosterol 14 alpha-demethylase binding with Pramiconazole (A), 12,28-Oxamanzamine A (B), Fascioquinol D (C), Saperconazole (D), Nakadomarin A (E), Plakinamine A (F), Fascioquinol C (G), Parsiguine (H), Hesperidin (I), Epoxyazadiradione (J)


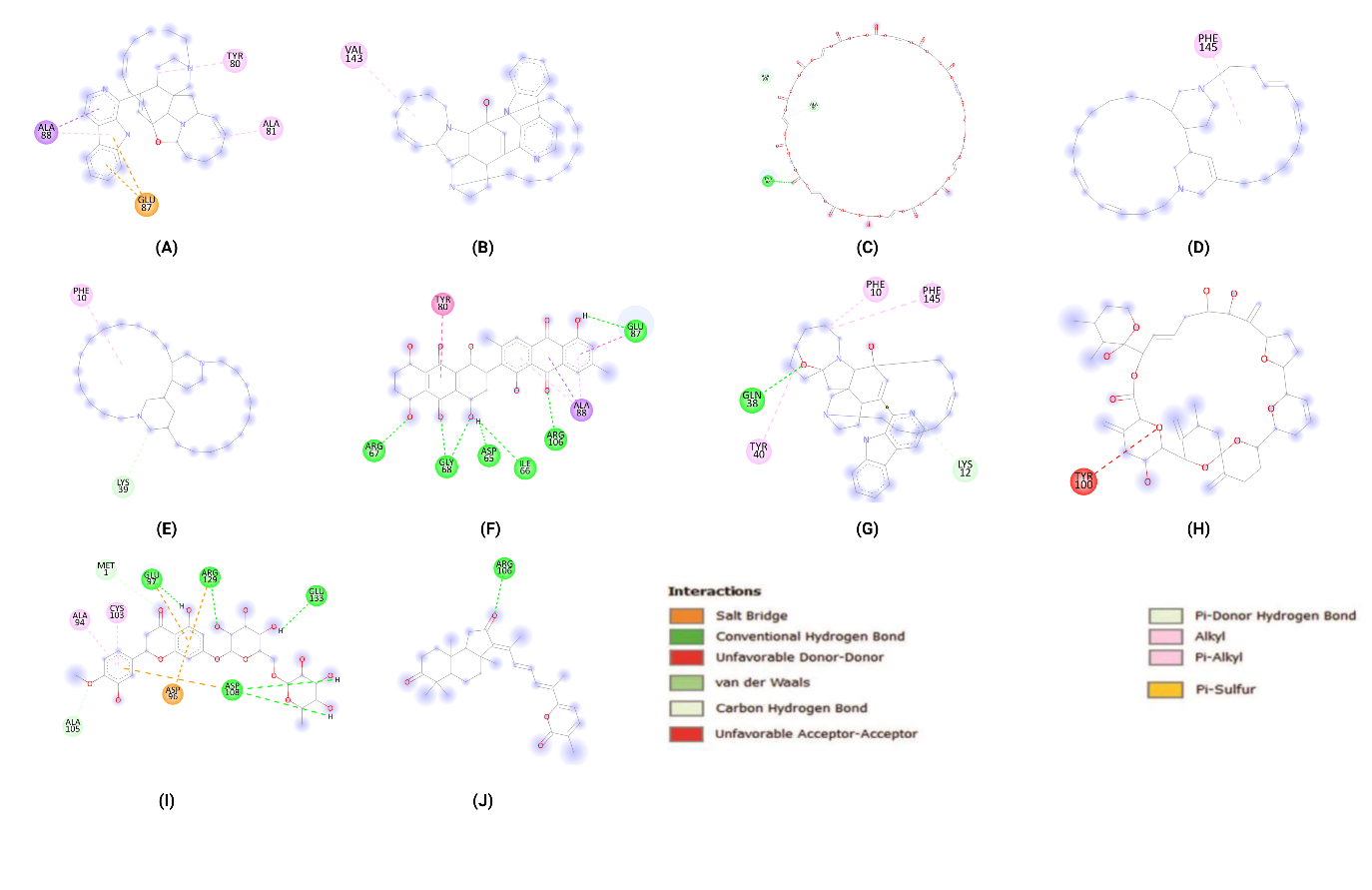


**Supplementary figure 3:** 2D visualization of docking analysis of Mucoricin binding with 12,28-Oxamanzamine A (A), Manzamine A (B), Parsiguine (C), Halicyclamine A (D), Tetrahydrohaliclonacyclamine A (E), Phaeosphenone (F), 6-Deoxymanzamine X (G), Goniodomin A (H), Hesperidin (I), Stelletin A (J).
