# Supplementary material for: In silico prediction and structure-based multitargeted molecular docking analysis of selected bioactive compounds against mucormycosis
Source: Bull Natl Res Cent. 2022 Jan 31;46(1):24. doi: 10.1186/s42269-022-00704-4 (PMC8802264; doi:10.1186/s42269-022-00704-4)
Supplement: Supplementary file 2 — Additional file 2: Table S1. The variants of mucormycosis and the symptoms associated with the disease (Kontoyiannis & Lewis, 2011; Ribes et al., 2000; Sheng, 2020; Spellberg et al., 2005; Symptoms of Mucormycosis | Mucormycosis | CDC, n.d.). [file 42269_2022_704_MOESM2_ESM.docx]

**Supplementary Table 1**. The variants of mucormycosis and the symptoms associated with the disease [1–5].

| Variant | Symptoms |
| --- | --- |
| Rhinocerebral | - One-sided facial swelling - Headache - Nasal or sinus congestion - Black lesions on the nasal bridge or upper inside of the mouth that quickly become more severe - Fever |
| Pulmonary | - Fever - Cough - Chest pain - Shortness of breath |
| Gastrointestinal | - Abdominal pain - Nausea and vomiting - Gastrointestinal bleeding |
| Cutaneous | - Blisters or ulcers - Infected area may turn black - Pain, warmth, excessive redness, or swelling |

**References:**

[1] J.A. Ribes, C.L. Vanover-Sams, D.J. Baker, Zygomycetes in Human Disease, Clin. Microbiol. Rev. 13 (2000) 236. https://doi.org/10.1128/CMR.13.2.236-301.2000.

[2] D.P. Kontoyiannis, R.E. Lewis, How I treat mucormycosis, Blood. 118 (2011) 1216–1224. https://doi.org/10.1182/blood-2011-03-316430.

[3] B. Spellberg, J. Edwards, Jr., A. Ibrahim, Novel Perspectives on Mucormycosis: Pathophysiology, Presentation, and Management, Clin. Microbiol. Rev. 18 (2005) 556. https://doi.org/10.1128/CMR.18.3.556-569.2005.

[4] W.H. Sheng, Epidemiology and clinical manifestations of influenza, J. Intern. Med. Taiwan. 31 (2020) 3–6. https://doi.org/10.6314/JIMT.202002_31(1).02.

[5] Symptoms of Mucormycosis | Mucormycosis | CDC, (n.d.).
