# Supplementary material for: In silico prediction and structure-based multitargeted molecular docking analysis of selected bioactive compounds against mucormycosis
Source: Bull Natl Res Cent. 2022 Jan 31;46(1):24. doi: 10.1186/s42269-022-00704-4 (PMC8802264; doi:10.1186/s42269-022-00704-4)
Supplement: Supplementary file 3 — Additional file 3: Data 1. Binding affinities of all bioactive compunds with our three target proteins. [file 42269_2022_704_MOESM3_ESM.pdf]

Supplementary Data 1. Binding affinities of all bioactive compunds with our three target proteins

S.no	Compounds	Pubchem CID	Binding affinity(kcal/ mol) with CotH3	Binding affinity(kcal/ mol) with Lanosterol 14 alpha-demethylase	Binding affinity(kcal/ mol) with Mucoricin
1	Sofosbuvir	45375808	-6.9	-8.4	-6.1
2	Ribavirin	37542	-6	-6.6	-5.3
3	5,7,4'-Trimethoxy-4-phenylcoumarin	12004232	-5.8	-7.6	-6.3
4	Eugenol	3314	-4.7	-5.9	-4.7
5	Natamycin	5284447	-8.7	-8.4	-6.9
6	Terbinafine	1549008	-6.4	-6.8	-5.2
7	Itraconazole	55283	-7.3	-9.5	-6.7
8	Posaconazole	468595	-7.8	-9.8	-6.4
9	Fluconazole	3365	-5.9	-7.5	-5.9
10	Isavuconazole	6918485	-6.5	-9.2	-5.9
11	Voriconazole	71616	-6.9	-7.4	-5.8
12	Pramiconazole	3013050	-7.6	-11	-7.1
13	Ravuconazole	467825	-5.7	-9.2	-6.3
14	Albaconazole	208952	-7.4	-9	-6.3
15	Zanamivir	60855	-6	-6	-5.2
16	Oseltamivir	65028	-5.1	-6.8	-5.2
17	Peramivir	154234	-5.6	-7.4	-5.6
18	3,4-Dimethyl-1,5-dihydro-2H-pyrrol-2-one	574037	-4.1	-4.6	-3.7
19	3-Epi-Xestoaminol C	90683233	-3.7	-5.5	-3.5
20	Xestoproxamine B	52937783	-7.6	-8.5	-6.5
21	Nakadomarin A	152772052	-7.6	-10.6	-6.5
22	Linalool	6549	-5.4	-5	-4.6
23	2,4-Dihydroxychalcone	6433293	-6.5	-8.1	-6.2
24	genistein	5280961	-6.9	-7.6	-6.2
25	Biochanin A	5280373	-6.4	-7.7	-6.2
26	Conocarpan	10999992	-7.3	-9.1	-6.8
27	beta-Eudesmol	91457	-6	-7.3	-5.9
28	thymol	6989	-6	-6.1	-4.8
29	Carvacrol	10364	-5.5	-6	-5.1
30	3,5 nonadiyne	185922	-4.5	-5.5	-4.4
31	alpha-Bisabolol	10586	-5.7	-7.2	-5.6
32	Ferutinin	91747167	-7.1	-8	-6.3
33	teferidin	10125942	-6.4	-8	-6.2
34	Borneol	64685	-5	-5.7	-4.3
35	FLUCYTOSINE	3366	-4.5	-5.2	-4.2
36	Stevensine	11003581	-6.2	-7.3	-5.5
37	Micafungin	477468	-6.5	-8.8	-6.2
38	Dalfopristin	6323289	-7.6	-7.8	-6.8
39	Ent-Manzamine F	6474731	-8.2	-8.4	-7.4
40	Olorofim	91885568	-8.6	-9.6	-7.4
41	Fosmanogepix	44123754	-7.7	-9.4	-6.6

42	Quercetin	5280343	-6.3	-8.3	-6.3
43	beta-Sitosterol	222284	-7.5	-8.7	-6.6
44	6-Deacetylnimbin	10505484	-7.1	-6.9	-6.5
45	Azadiradione	12308714	-6.9	-8.6	-6.4
46	Nimbin	108058	-6.2	-6.8	-5.9
47	Salannin	6437066	-6.9	-6.8	-5.8
48	Epoxyazadiradione	49863985	-7	-9.9	-6.2
49	Nimonol	73356511	-6.3	-7.7	-6.3
50	Meliantriol	46173826	-7.5	-8.7	-6.3
51	Allicin	65036	-3.4	-3.9	-3.2
52	Allyl isothiocyanate	5971	-3.3	-3	-2.5
53	S-Methyl methanesulfinothioate	95200	-2.7	-3	-2.5
54	Diallyl disulfide	16590	-3	-3.6	-3.2
55	Ajoene	5386591	-3.6	-4.3	-3.6
56	2-Vinyl-4H-1,3-dithiine	133337	-4.5	-4.1	-3.4
57	3-Vinyl-4H-1,2-dithiin	150636	-4.7	-4.4	-3.4
58	Phlorofucofuroeckol A	130976	-8	-8.9	-7.5
59	7-O-Methylkoninginin D	46833400	-6.2	-7	-5.4
60	Geraniol	637566	-4.5	-5.5	-4.3
61	Sordarin	485851	-6.7	-7.1	-6.1
62	Griseofulvin	441140	-5.7	-7	-5.9
63	morpholine	8083	-3.5	-3.4	-3.2
64	Nikkomycin z	456557	-7	-7.9	-6.2
65	Papulacandins	6450326	-7	-8.6	-5.6
66	Stelletin A	5352083	-7.4	-8.2	-7.8
67	Cycloeudesmol	442362	-5.7	-6.8	-5
68	laurinterol	11471955	-6.9	-7.2	-5.7
69	Debromolaurinterol	466442	-6.8	-7.7	-5.8
70	parsiguine	153274516	-9.2	-10.1	-8.2
71	Elatol	479931	-5.5	-5.9	-5.1
72	Karatungiol	101397831	-5.2	-6.6	-4.1
73	9H-Pyrido[3,4-B]indole	64961	-7.4	-6.5	-5.5
74	Deschloroelatol	479932	-5.8	-7.9	-5.1
75	Loliolide	100332	-6.8	-6.5	-4.9
76	beta-Cyclocitral	9895	-4.7	-5.6	-4.3
77	Neophytadiene	10446	-3.8	-5.2	-4.3
78	Lasiodiplodin	14562696	-5.9	-7.9	-5.5
79	Tolnaftate	5510	-7.1	-7.9	-6.6
80	Undecylenic acid	5634	-4.1	-5	-4
81	Miconazole	4189	-6.7	-7.9	-6.1
82	Clotrimazole	2812	-6.9	-8.3	-5.8
83	Sclerotiotide A	46849843	-5.9	-7.1	-6.2
84	Butenafine	2484	-7.6	-7.8	-5.7
85	Bifonazole	2378	-8	-9.5	-6.9
86	Terconazole	441383	-7.2	-8.7	-6.9
87	Ciclopirox	2749	-5.4	-6.4	-5
88	Haloprogin	3561	-4.3	-4.2	-4
89	Tioconazole	5482	-6.3	-7.2	-5.6
90	Miltefosine	3599	-4.7	-4.9	-4.3

91	Sulconazole	5318	-7.1	-7.2	-6.1
92	Ketoconazole	456201	-7.1	-8.5	-6.3
93	Manzamine A	6509753	-8.1	-9	-8.3
94	Efinaconazole	489181	-7.1	-7.6	-5.6
95	Butoconazole	47472	-6.4	-7.3	-5.7
96	Dimazole	8708	-4.9	-6.6	-4.5
97	Manoalide	6437368	-7	-8.6	-6.2
98	Chloroxine	2722	-5.5	-6.1	-5.1
99	Oxiconazole	5353853	-6.5	-7.4	-6.2
100	Xestodecalactone B	9943580	-6.9	-6.7	-6.2
101	Chloroxylenol	2723	-5	-5.5	-4.6
102	Amorolfine	54260	-6.3	-7.2	-6.2
103	T-2307	9803135	-6.4	-7.5	-5.2
104	Econazole	3198	-6.7	-7.9	-6.1
105	Clove oil	105755	-5.8	-6.8	-5.3
106	Fleroxacin	3357	-6.4	-7.4	-6.1
107	Pyrithione	1570	-4.7	-4.4	-3.7
108	Vialinin B	16049791	-8.9	-7.8	-6.5
109	Levoketoconazole	47576	-7.5	-8.5	-6.8
110	Hexetidine	3607	-5.4	-6	-4.7
111	Hydroxystilbamidine	5284571	-6.5	-7.6	-6.1
112	Methylchloroisothiazolinon e	33344	-4.6	-4.3	-3.2
113	Quilseconazole	91886002	-7.7	-8.1	-7.6
114	Isavuconazonium	6918606	-4.7	-7.8	-6.4
115	Pentamidine	4735	-6.3	-5.6	-5.1
116	Dipyrithione	3109	-5.8	-6.9	-5.4
117	Salicylic acid	338	-6.4	-5.4	-4.6
118	Clioquinol	2788	-5.2	-5.9	-5.1
119	Naftifine	47641	-6.3	-7.8	-6
120	Gentian Violet	11057	-6.5	-7.5	-6.4
121	Isoconazole	3760	-6.5	-8	-5.8
122	Sertaconazole	65863	-7	-7.7	-6.5
123	Luliconazole	3003141	-5.8	-7.8	-5.5
124	Streptomycin	19649	-6.7	-6.4	-5.7
125	Dequalinium	2993	-7.1	-6.9	-5.9
126	Chlorophetanol	15907	-4.1	-4.8	-4.4
127	Potassium iodate	23665710	-4.4	-4.4	-3.9
128	Capric acid	2969	-4.1	-4.6	-4
129	Benzethonium	2335	-5.4	-7.6	-4.7
130	Oteseconazole	77050711	-7.7	-8.6	-6.9
131	VT-1598	126715974	-6.6	-7.5	-6.3
132	Bafilomycin A1	6436223	-7	-7.9	-6.4
133	Allosamidin	119339	-5.8	-7.7	-6.1
134	Salicylhydroxamic Acid	66644	-6.7	-6	-5.2
135	Trichostatin A	444732	-7.2	-6.8	-5.5
136	Pyroquilon	91665	-5.2	-6.5	-4.9
137	Abafungin	159326	-7.7	-7.9	-6.7
138	Cerulenin	5282054	-4.9	-5.5	-4.8
139	Pyrrolnitrin	13916	-5.3	-6.5	-5

140	Triclosan	5564	-5.1	-5.8	-5.7
141	Triacetin	5541	-4.4	-5.3	-4.2
142	Pentamycin	5282200	-6.3	-8.2	-6.2
143	Triclocarban	7547	-6.1	-8	-6.3
144	Radicicol	6323491	-6.9	-8.7	-6.6
145	Pecilocin	5282176	-5.8	-7	-5.8
146	Troclosene	16726	-5.1	-5.8	-4
147	Tolciclate	5506	-6.9	-9	-6.2
148	Nitroxoline	19910	-6.5	-6.5	-4.8
149	Icofungipen	216240	-7.4	-9.2	-6.4
150	Sinefungin	65482	-7.2	-6.7	-6
151	Myxothiazol	10972974	-6	-6.4	-5.6
152	Flutrimazole	3401	-6.7	-8.1	-5.8
153	Cordycepin	6303	-5.3	-6.5	-5.4
154	Lys-Nva-FMDP	6475796	-5.7	-5.9	-5.4
155	Fenticonazole	51755	-7	-8.7	-6.6
156	Omoconazole	3033988	-6.4	-6.4	-6.4
157	Lawsone	6755	-5.5	-6.9	-5.6
158	Nifuratel	6433427	-5.3	-6.3	-5.4
159	Mevastatin	64715	-6.8	-7.4	-6.1
160	Wortmannin	312145	-6.8	-8.6	-6.1
161	Tea tree oil	22833361	-3.6	-4.4	-3.9
162	Flindersine	68230	-6.4	-8	-6.2
163	syn-2869	490657	-8.2	-9.4	-7
164	5-Epi-Ilimaquinone	21727418	-6.7	-8.7	-6
165	Azoxystrobin	3034285	-6.7	-7	-6
166	Fumagillin	6917655	-5.9	-6.9	-5.7
167	Motualevic acid A	25242622	-5.5	-6.2	-4.4
168	Agelasine A	10072839	-7.7	-7.7	-6.2
169	Agelasine B	6439899	-7.9	-9.1	-6.5
170	(+)-Kalihinol A	185105	-7	-8.9	-6.1
171	Phyllofenone B	23426940	-7.7	-8.2	-7.4
172	Moxifloxacin	152946	-7.2	-7.2	-6.2
173	Eurysterol A	23706901	-6.5	-7.7	-6.2
174	5alpha-Cholestane	2723895	-6.8	-8.5	-6.3
175	Eurysterol A sulfonic acid	16681436	-6.6	-7.5	-6.2
176	Eurysterol B	23706902	-6.7	-9.4	-6.6
177	Eurysterol B sulfonic acid	16681438	-7.3	-9.3	-6.6
178	Geodisterol-O-sulfite	44254699	-7.5	-8.6	-6.8
179	Heteronemin	21589810	-7.6	-7.4	-6.6
180	Heteronemin acetate	21589811	-8	-8.1	-6.5
181	Fascioquinol A	53233477	-8.2	-9.3	-6.8
182	Benzosceptrin C	44598334	-7.4	-9.1	-6.9
183	Chebulic acid	71308174	-6	-6.2	-5.7
184	Oroidin	6312649	-5.7	-6.3	-5.3
185	Bromoageliferin	15816051	-6.7	-7.8	-6.5
186	Alisiaquinol	24970869	-7.5	-8.7	-7
187	Alisiaquinone A	24970867	-7.3	-9.1	-7
188	Plakinamine A	21606893	-7.7	-10.4	-7.2

189	Ceratinadin A	49863115	-7.9	-8.9	-6.7
190	Fasciospongine A	16755903	-6.6	-9	-6.5
191	Haliclonin A	101462407	-7.7	-8.6	-6.7
192	Halicyclamine A	10479489	-7.8	-9.1	-8.2
193	Haliclonacyclamine A	102423951	-7.8	-8.7	-6.9
194	Ethylhexylglycerin	9859093	-4.4	-5	-4.2
195	oceanapamine	10313635	-6.2	-6.8	-5.4
196	Topsentin	72457	-8.1	-9.7	-7.1
197	Nephalsterol B	463812	-6.6	-7.7	-6.3
198	Aerothionin	10919908	-6.9	-7	-6.4
199	(-)-Agelasidine C	5467446	-6.7	-7.3	-5.7
200	Goniodomin A	6440832	-7.7	-9.5	-8
201	Trichoderin A	46891555	-6.2	-6.4	-4.7
202	Demethoxycurcumin	5469424	-6.7	-7	-5.4
203	Bisdemethoxycurcumin	5315472	-6.7	-7.6	-6.6
204	(5R,6R)-7,9-Dibromo-6-hydroxy-8-methoxy-N-[2-[1-(3,5,8-trihydroxy-4-oxo-1H-quinolin-6-yl)imidazol-4-yl]ethyl]-1-oxa-2-azaspiro[4.5]deca-2,7,9-triene-3-carboxamide	46911279	-8.2	-9	-6.5
205	Ceratinadin B	49863116	-7.6	-8.9	-7.6
206	Ceratinadin C	49863117	-5.7	-7.7	-6.1
207	Avarol	72185	-6.8	-7.3	-6.6
208	Avarone	72186	-7.1	-8.4	-6.4
209	Fascioquinol E	10600155	-6.8	-7.2	-5.6
210	Fascioquinol C	53328564	-7.9	-10.4	-7.2
211	Fascioquinol F	67831571	-7.4	-8.3	-6.4
212	Fascioquinol B	53233589	-7.1	-9.5	-6.7
213	Fascioquinol D	53328565	-7.8	-10.8	-7.2
214	Topsentin D	12018820	-7.5	-9.5	-7.1
215	Bromotopsentin	183528	-8	-9.3	-7.2
216	Topsentine	44416636	-7.8	-9.1	-7.7
217	Deoxytopsentin	183527	-8.5	-9.5	-7.2
218	Woodylide A	71720654	-5.7	-6.3	-4.7
219	Clathric acid	102486003	-6.7	-7.2	-5.9
220	Metachromin A	10473589	-6.6	-7.7	-6.2
221	Mamanuthaquinone	11969939	-6.7	-7.6	-5.8
222	Hymendin	6439099	-5.9	-6	-5.4
223	Palauolide	13859138	-7.7	-8.2	-7
224	Clathrodin	5388709	-5.7	-6.2	-5.3
225	Sventrin	10938301	-5.8	-7.1	-5.4
226	Stachybogrisephenone B	139583964	-7.2	-6.9	-6.1
227	Latrunculin B	6436219	-7	-9.3	-6.5
228	Tirandamycin A	54706137	-6.6	-8.4	-6.8
229	Kuchinoenamine	136870410	-6.4	-8	-5.7
230	Botryorhodine D	42637419	-6.4	-7.8	-6.2
231	Nakijiquinone H	44586960	-6.7	-7.1	-6.4

232	Smenospongine	3081931	-7.1	-7.6	-6.8
233	Saperconazole	457278	-7.8	-10.8	-7
234	Ent-Chromazonarol	11674059	-7.3	-8.7	-6.7
235	Conckurchine	76330309	-7.2	-9	-6.9
236	cis-3,4-Dihydrohamacanthin B	10624851	-8	-9.3	-6.9
237	Isoquercitrin	5280804	-7.4	-8.3	-6.1
238	Haliclonacyclamine C	102124133	-8	-7.4	-6.9
239	(+)-Agelasine D	11775481	-7.8	-8.6	-6
240	9-Methyl-7-[(E)-3,7,11,15-tetramethylhexadec-2-enyl]purin-7-ium-6-amine	44429795	-6.1	-6.8	-5.1
241	Tjipanazole A1	10050435	-7.6	-8.1	-7
242	Hesperidin	10621	-8	-10	-8
243	Piperine	638024	-7.2	-8.4	-6.3
244	Rutin trihydrate	16218542	-7.2	-8	-6.6
245	Caryophyllene oxide	1742210	-5.6	-7.3	-5.3
246	Riboflavin	493570	-6.7	-6.3	-6.1
247	Folic Acid	135398658	-7.1	-8.7	-6.6
248	Murrayanine	96942	-6	-7.3	-5.7
249	Isomurrayafoline B	375146	-6.5	-7.5	-6
250	Girinimbine	96943	-6.9	-9.2	-6.6
251	Tetrahydrohaliclonacyclamine A	46894059	-8.2	-8.2	-8.2
252	2-[[2-[[2-(2,6-Diaminohexanoylamino)-3-(4-hydroxyphenyl)propanoyl]amino]-3-(1H-imidazol-5-yl)propanoyl]amino]propanoic acid	18309602	-6.3	-6.2	-5.7
253	(2R,4S)-2-Amino-4-[[4-[(2-amino-4-oxo-1H-pteridin-6-yl)methylamino]benzoyl]amino]-2-[(4-hydroxy-3,5-diiodophenyl)methyl]-3-oxoheptanedioic acid	136023163	-6.7	-6.8	-6.8
254	Halicyclamine B	10628970	-7.5	-6.6	-6.6
255	Neopterin	135408877	-6.4	-5.6	-5.1
256	Pteroic acid	135398749	-6.1	-6.2	-6.4
257	2 Deoxy D glucose	108223	-4.9	-4.3	-4.1
258	Neopetrosiamine A	49765082	-7.4	-6.7	-6.7
259	Haliclonacyclamine B	102124134	-9.2	-6.9	-6.9
260	(GlcNAc)2 (Man)3 (Xyl)1	71297386	-6	-6	-5.8
261	Cudraxanthone S	5495918	-6.8	-8.3	-6.6
262	Cudraflavanone B	509244	-6.5	-7.7	-6.3
263	Cupressotropolone A	10087743	-6.4	-7.5	-6.1
264	Cupressotropolone B	10001595	-6.6	-7.8	-6

265	1-[2′,3′-dihydroxy-5′-(hydroxymethyl)phenyl]-3-methyl-but-2-ene	9990627	-5.7	-6.5	-5.4
266	Moschamine	5969616	-7.1	-8.1	-6.5
267	5-(8’-Z-heptadecenyl) resorcinol	53774196	-5.2	-5.7	-4.6
268	Buddledin A	5281514	-5.9	-7	-5.4
269	Buddledin B	6443194	-5.6	-7.3	-5.5
270	Pestafolide A	24850054	-6.5	-7.7	-6
271	Pestaphthalide A	24850093	-5.6	-6.7	-5.1
272	Pestaphthalide B	24850094	-6.1	-6.3	-5
273	Phaeosphenone	24970762	-8	-8.8	-8.2
274	4-Terpenyl Cannabinolate	24850050	-7.4	-7.9	-6.3
275	4-(Furan-3-yl)benzaldehyde	21526045	-5.5	-6.1	-4.9
276	4-(5-oxotetrahydrofuran-3-yl)benzaldehyde	14863055	-6.9	-6.8	-5.3
277	Solanapyrone J	23625389	-6.1	-7.9	-6
278	8alpha-O-(4-Hydroxy-2-methylenebutanoyloxy)-11beta,13-dihydro-4-epi-sonchucarpolide	24178999	-6.4	-7.7	-5.8
279	8alpha-O-(4-Hydroxy-2-methylenebutanoyloxy)-11beta,13-dihydrosonchucarpolide	24178998	-6.9	-7.4	-5.9
280	Neopeltolide	16115403	-6.6	-7.1	-6.1
281	Gentirigeoside A	44422964	-6.6	-7.1	-6.8
282	(-)-Rotiorin	11574449	-7.4	-7.5	-6.1
283	Anthecotulide	11962174	-4.9	-6.9	-5.4
284	acetoxyanthecotulide	11522381	-5.6	-5.9	-5.6
285	6-Epi-oxysporidinone	54677669	-6.9	-6.9	-6.1
286	Andrographatoside	11533372	-6.2	-7.8	-6
287	Latrunculin T	15939613	-5.5	-6.6	-4.9
288	Carneic acid A	16083152	-6.5	-7.3	-5.8
289	Carneic acid B	16083153	-6.6	-7.2	-5.9
290	Cinnamodial	442354	-5.3	-6.9	-5.1
291	Cinnamosmolide	12303262	-6.7	-6.5	-5.5
292	Resorstatin	198749	-5.8	-6.5	-5.1
293	Rotiorinol A	11617875	-6.3	-8.6	-6.1
294	Epi-isochromophilone II	11661102	-7.2	-7.9	-6.1
295	Chrysotrione A	16104929	-5	-5.8	-4.8
296	Manzamine E	44445401	-8.3	-8.8	-7.8
297	Musanolone C	10804574	-6.9	-8.1	-7.3
298	Andrographolide	5318517	-6.8	-8	-6.1
299	6-Deoxymanzamine X	44445400	-8.8	-9.2	-8.1
300	12,28-Oxamanzamine A	11272782	-10.2	-10.9	-8.6
